# Carbon Capture Utilization and Storage in Methanol Production Using a Dry Reforming-Based Chemical Looping Technology

**DOI:** 10.1021/acs.energyfuels.2c00620

**Published:** 2022-07-19

**Authors:** Ambrose Ugwu, Mogahid Osman, Abdelghafour Zaabout, Shahriar Amini

**Affiliations:** † Department of Energy and Process Engineering, Norwegian University of Science and Technology, 7034 Trondheim, Norway; ‡ Process Technology Department, SINTEF Industry, 7034 Trondheim, Norway; § Department of Mechanical Engineering, The University of Alabama, Tuscaloosa, Alabama 35487, United States

## Abstract

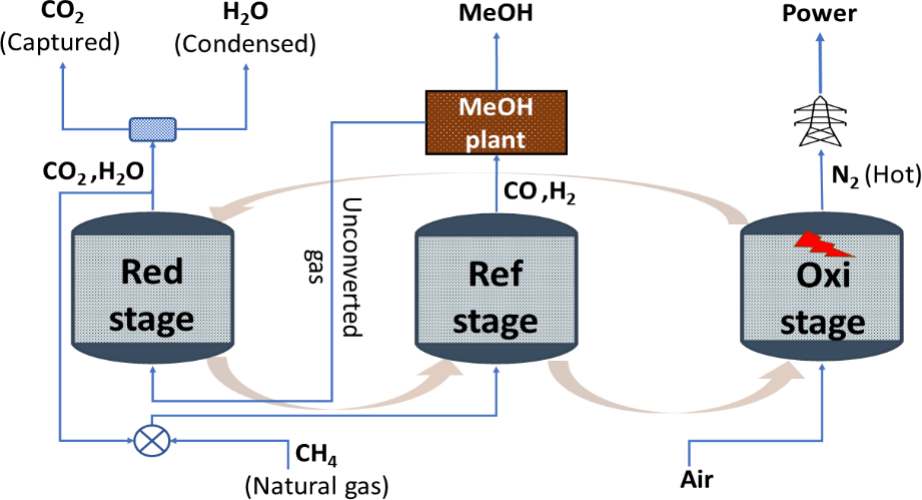

This further investigates the concept of gas switching dry reforming (GSDR) that efficiently converts the two major greenhouse gases (CO_2_ and CH_4_) into a valuable product (syngas) for gas-to-liquid (GTL) syntheses. The proposed GSDR is based on chemical looping technology but avoids external circulation of solids (metal oxides) by alternating the supply of reducing and oxidizing gas into a single fluidized bed reactor to achieve redox cycles. Each cycle consists of three steps where a metal oxide/catalyst is first reduced using GTL off-gases to produce CO_2_ (and steam) that is supplied to the next reforming step to produce syngas for GTL processes. The metal oxide is then reoxidized in the third step associated with heat generation (through the exothermic oxidation reaction of the metal oxide and air) to provide the heat needed for the endothermic dry methane reforming step. Experimental demonstrations have shown that a syngas H_2_/CO molar ratio between 1 and 2 suitable for methanol production could be achieved. A further demonstration shows that pressure has negative effects on gas conversion. Following the successful experimental campaign, process simulations were completed using ASPEN to show how the GSDR process can be integrated into a methanol (MeOH) production plant.

## 1 Introduction

**Figure 1:**
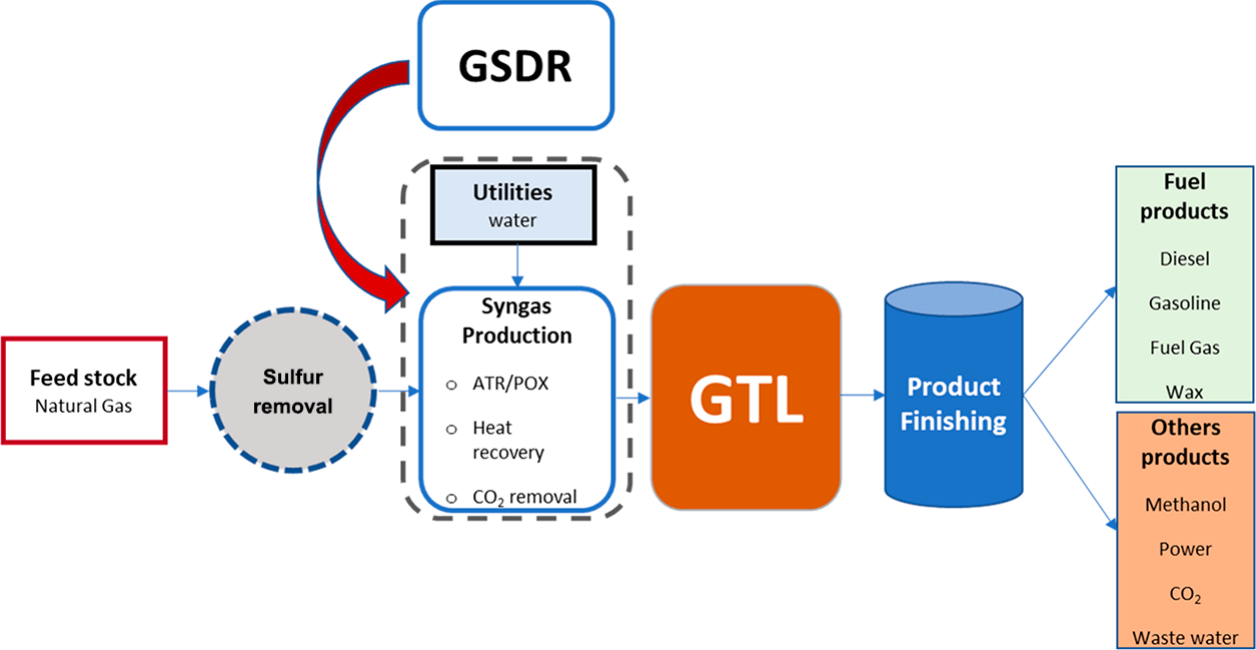
Illustration of a state-of-the-art Fischer–Tropsch process by U.S. Energy Information Administration^3^ with the possibility of GSDR–GTL integration.

The conventional methods to convert natural gas in GTL processes are energy intensive and usually associated with high investment costs to handle harsh process conditions.^1,2^ A state-of-the-art Fischer–Tropsch process proposed by the U.S. Energy Information Administration (Figure 1) can be used for illustration.^3^ This scheme shows that several steps are needed^3^ to convert natural gas to the final liquid fuel product where the syngas (a mixture of H_2_, CO, and CO_2_) production step is the most complex, energy demanding, and cost intensive.^4,5^ The produced syngas is then sent to the GTL reactor where H_2_ and CO combine to produce the liquid hydrocarbons of interest (Reactions 1–4).^6,7^ The liquid products are further sent to other refinement steps (such as thermal cracking) to obtain the desired product specification and separate the unconverted syngas.

**Figure 2:**
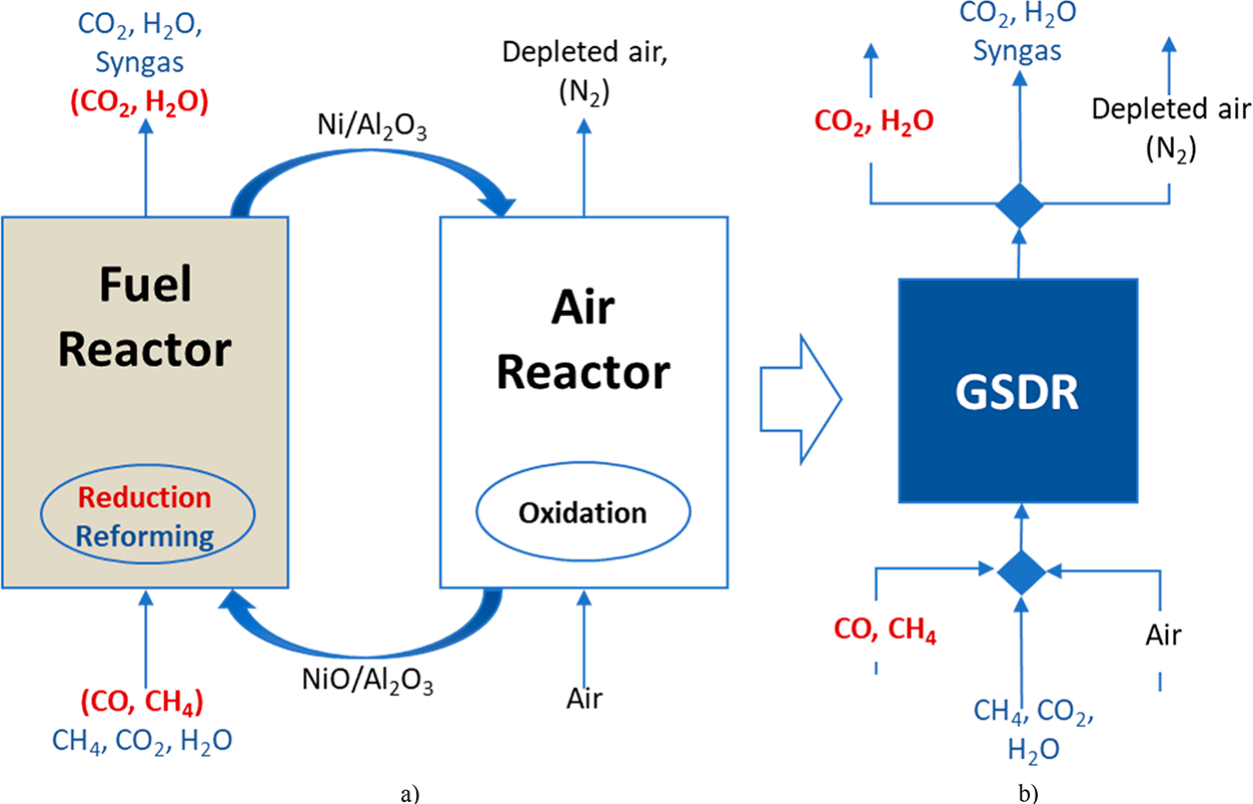
Conceptual schemes of the dry reforming process: (a) conventional chemical looping approach and (b) gas switching dry reforming (GSDR) approach.^11^

At a commercial scale, autothermal reforming of natural gas with an integrated air separation unit is usually applied for syngas production when targeting GTL production. This approach has not received many economic benefits due to the high cost of the air separation unit and the associated CO_2_ emissions if the source of electricity for powering the latter is not renewable.^8^ This prompted research in alternative reactor designs (e.g., microchannel reactor) and catalyst development.^9,10^ Among these reactor designs, gas switching dry reforming (GSDR) with integrated carbon capture and utilization (as illustrated in Figure 1) has been proposed to replace the conventional syngas production step.^11^ The gas switching technology is based on chemical looping technology which has been proven to be highly efficient, economical, and more environmentally friendly compared to other existing technologies.^12,13^ Extensive research have been completed on this novel technology concept from process modeling, material development, and scaled up from lab scale to a prepilot scale.^14^ Schematic illustrations of the conventional chemical looping and the proposed GSDR approaches for syngas production from CH_4_, and CO_2_ (dry methane reforming) are depicted in Figure 2. The cycle comprises three steps where a metal oxide (oxygen carrier/catalyst) is circulated between interconnected fluidized bed reactors (air and fuel reactors shown in Figure 2a for the conventional configuration) to acquire and release oxygen (lattice oxygen), thus avoiding the need for an air separation unit (ASU).^8,15^ In the fuel reactor, the metal oxide is reduced to the metallic state by the reaction between the fuel and the metal oxide in a N_2_-free environment to produce pure CO_2_ or a mixture of CO_2_ and H_2_O which is captured and utilized in the reforming (syngas production) step. The reduced oxygen carrier serves as a catalyst to speed up the methane reforming reaction (steam/dry). The quality of the produced syngas in this stage is controlled to the suitable H_2_/CO molar ratio to match the necessary downstream reaction conditions, with minimal undesirable products. The reduced metal oxide is then regenerated in the air reactor by reacting with oxygen (from air). The oxidation reaction with air is highly exothermic which generates the heat needed for the endothermic (reduction and reforming) reactions taking place in the fuel reactor.

The switching nature of the proposed GSDR concept offers additional benefits where the oxidizing and reducing gases are alternated in a single fluidized bed^16,17^ to avoid external solids circulation thus simplifying the pressurizing of syngas production close to the pressure of the downstream GTL process (Figure 2b).^18,19^ Additionally, GSDR can be operated autothermally^11^ to utilize the unconverted GTL off-gases in a separate reduction step, while the outlet gases from the reduction step (consisting of CO_2_, H_2_O, and unconverted CH_4_) can be fed to the reforming step with additional CH_4_ to produce syngas for a GTL downstream process. Such integration maximizes fuel utilization and eliminates CO_2_ emissions. More importantly, since syngas quality (H_2_/CO molar ratio) is one of the most important factors that determines the outcome of a GTL process,^20^ a process such as GSDR that offers the flexibility to control this parameter to suit different GTL applications is of great interest. The high H_2_/CO molar ratio of ≥ 3 from steam methane reforming and low H_2_/CO molar ratio of ≤ 1 from dry methane reforming are not optimal for the GTL processes.^21,22^ To modify the H_2_/CO molar ratio to the optimal value, CO_2_ utilization in the reforming process has been proposed in several studies^11,23^ including chemical looping which makes the dry reforming of natural gas more economically and environmentally attractive. With the chemical looping option, the possible reactions at the syngas production step could vary from Reactions 7 to 13 (Table 1).

**Table 1:** Different Reaction Schemes

GTL Reactions	
Methanation	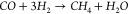 1
Paraffins	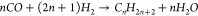 2
Olefins	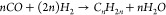 3
Alcohols	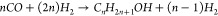 4

GSDR Reactions	
Reduction stage	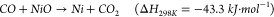 5
	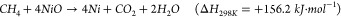 6
Reforming stage	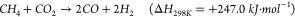 7
	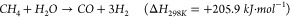 8
	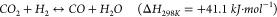 9
	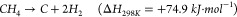 10
	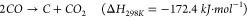 11
	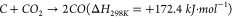 12
	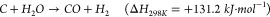 13
Oxidation stage	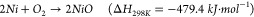 14
	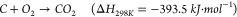 15
	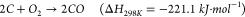 16

Previous studies have demonstrated that it is possible to adjust the syngas quality (H_2_/CO molar ratio) of the dry reforming reaction (Reaction 7)^24,25^ to an optimal value suitable for GTL processes,^26^ which motivated the first study of autothermal gas switching dry reforming (GSDR).^11^ The novel GSDR approach could also prevent the problem of catalyst deactivation through carbon deposition (a major drawback of conventional dry reforming), benefiting from the cyclic gasification of the deposited carbon (Reactions 10 and 11) in the oxidation stage, however at the expense of reduced CO_2_ capture efficiency.^11^ A steam gasification step could instead be implemented just after the dry reforming to remove the deposited carbon in the form of additional syngas that could be fed to the downstream GTL process.

Carbon deposition mainly from methane cracking led to a very high H_2_/CO molar ratio (≫1, imposing the need for larger recycling that increases cost for GTL integration. Feeding larger CO_2_/CH_4_ was found to minimize carbon deposition but leads to a very low syngas ratio (H_2_/CO molar ratio < 1).^11^ Song et al. suggested steam addition in the reforming stage to achieve a combined dry and steam reforming effect to tune the syngas ratio (H_2_/CO molar ratio) and reduce carbon deposition^27^ in agreement with conclusions from a previous study.^28^ Recently, Lee et al. adopted a similar approach using a membrane reactor and could control the H_2_/CO ratio by manipulating the CH_4_/CO_2_/H_2_O input ratio.^29^

With the aforementioned challenges, this study experimentally explores different options to improve the syngas quality (H_2_/CO ratio) of the GSDR process proposed in our previous study.^11^ This is to facilitate the integration of GSDR to GTL processes and enable better control of GTL products at reduced process steps. Two main approaches were adopted to improve the H_2_/CO ratio: (i) tuning of the CO_2_:CH_4_ molar ratio in the gas feed and (ii) substitution of part of the CO_2_ feedstock with steam. A sensitivity study at elevated pressure up to 5 bar was completed to highlight the effect of pressure on the H_2_/CO ratio. The responses of other key performance indicators, such as feed gas conversion, both in the reduction and reforming stages, and carbon deposition, to the different operating parameters, were investigated. Finally, process simulations were completed to evaluate how the proposed GSDR process can be integrated into a methanol production plant.

## 2 Experiment Demonstration

### 2.1 Experimental Setup

**Figure 3:**
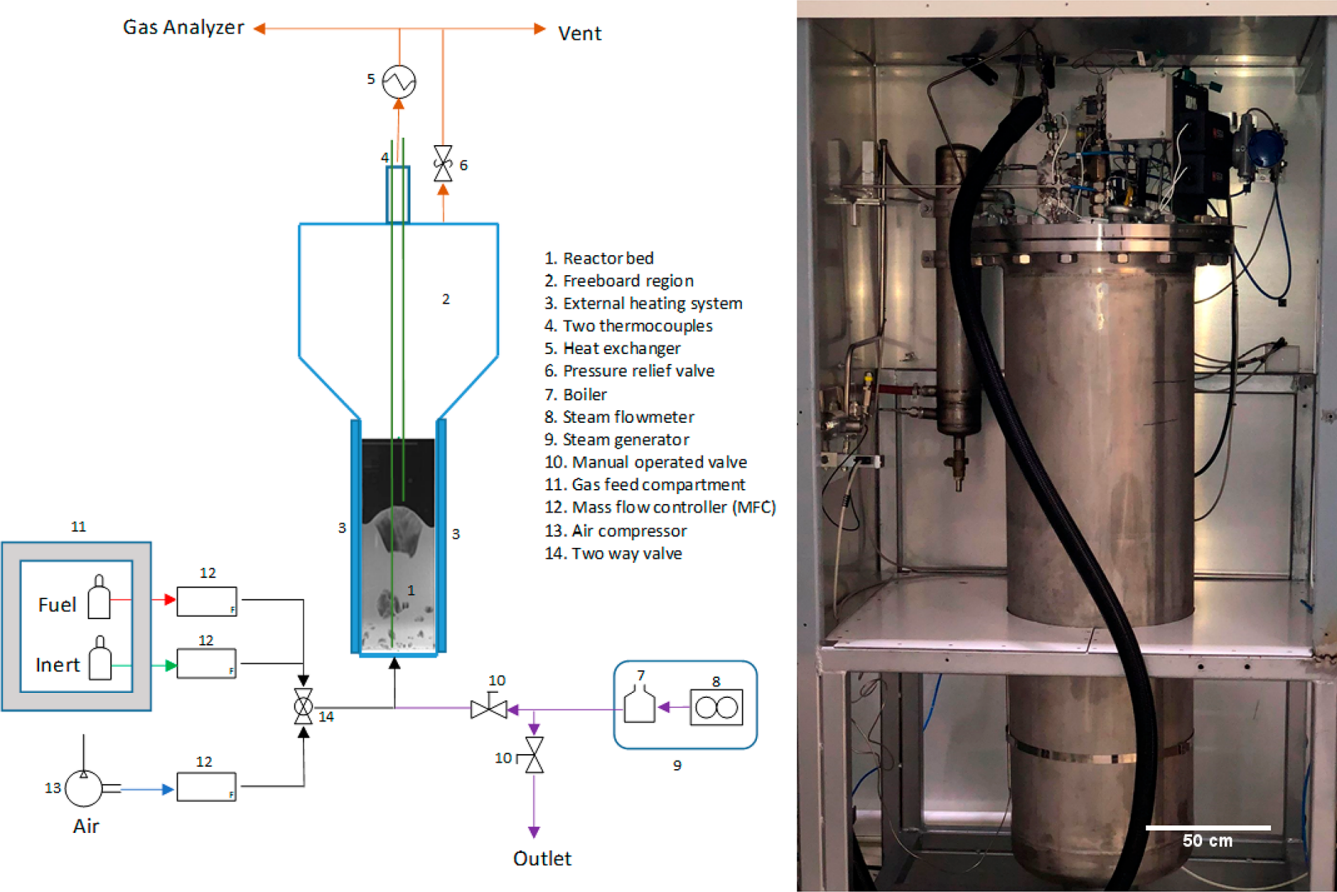
Standalone gas switching reactor used for GSDR experiments.

The experimental setup consists of a fluidized bed reactor with a 5 cm inner diameter and 50 cm height, in addition to a freeboard region, expanding from 5 to 10 cm ID at the top, to minimize particle entrainment (Figure 3). The setup has been used for previous studies^30,31^ with a reactor height of (including the body and the freeboard) of 90 cm. The reactor is made of Inconel 600 to withstand high temperatures up to 1000 °C. Gas is fed into the reactor using a lance extending toward the bottom of the reactor to create a fountain for effective gas distribution. Heat is supplied to the reactor through external electrical heating elements wound around the reactor vessel and covered with a 25 cm thick insulation. The control of the process parameters, data acquisition, and logging is done through a LabVIEW application. Bronkhorst mass flow controllers were used to regulate the gas feed into the reactor. A three-way valve separates the air and fuel feeds during the redox process. The outlet gas stream is passed through a cooler to reduce the temperature to the acceptable level for the gas analyzer and the ventilation system. An ETG syngas analyzer is used to measure the gas composition, while the temperature is measured using two thermocouples located at 2 and 20 cm inside the bed.

### 2.2 Materials and Method

This experimental study was completed using a NiO/Al_2_O_3_ oxygen carrier with 35% active content. About 623 g of the oxygen carrier was used corresponding to a 0.3 m static bed height. The particle size cutoffs D_10_, D_50_, and D_90_ are 117.4, 161.7, and 231.3 μm, respectively. The loosely packed density is 1950 kg/m^3^, while the tapped density is 2166 kg/m^3^. This oxygen carrier has been used in the first autothermal demonstration of the GSDR process^11^ and also used for other previous chemical looping studies including combustion^32,33^ with good stability and catalytic performance.

Typical GSDR cycles were completed starting with the reduction stage by feeding a gaseous fuel (CO/CH_4_) to react with NiO to produce Ni which catalyzes the reforming reaction. Note that the GTL off-gases consist mainly of a mixture of CO, H_2_, and CH_4_ (in addition to CO_2_) which converts well with the oxygen carrier in the reduction reactions,^34^ making GSDR–GTL integration a feasible option. The reduction stage is followed by the reforming stage where CH_4_ and CO_2_/H_2_O are cofed in the presence of Ni (catalyst) to produce syngas (CO and H_2_) through reforming reactions. This stage is energy demanding, justifying the need for the consecutive exothermic oxidation stage where pure air is fed to oxidize Ni back to NiO to produce heat for the process and regenerate the oxygen carrier, in addition to removing any deposited carbon on the catalyst from the precedent stages.

All the experiments were performed at an average temperature of 850 °C maintained through a combination of the heat of reactions and external electrical heating. For the experiments at atmospheric conditions, 12.8 NLPM CO was fed into the reactor for 3 min to achieve 50% oxygen carrier utilization at the reduction stage. Also, 3.2 NLPM CH_4_ (and CO_2_ at various CH_4_/CO_2_ ratios) was fed in the reforming stage, and 10 NLPM feed of pure air was fed in the oxidation stage. To achieve good mixing and optimal heat transfer, the gas flow was maintained within the bubbling/turbulent fluidization region. Temperature, pressure, and gas composition readings were recorded, and the reactor performance was evaluated using the measures as presented in Section 2.2.1.

#### 2.2.1 Reactor Performance Indicators

Different indicators have been defined in this section to evaluate the GSDR reactor performance bearing in mind that the objective of the GSDR process is to convert CH_4_ and CO_2_/H_2_O to syngas (H_2_ and CO) with minimal CO_2_ emission. It is desired to have maximal fuel conversion in the reduction stage to produce a pure stream of CO_2_ and maximal CH_4_, CO_2_, and H_2_O conversion in the reforming stage, respectively. At the reduction stage, the CO conversion is important since it determines how much Ni would be available to catalyze the reforming reactions and quantified in eq 17. At the reforming stage, the syngas quality H_2_/CO molar ratio (eq 18) is very important for integration to GTL processes, while CH_4_, CO_2_, and H_2_O conversions determine the extent of the reforming reaction, selectivity, and overall syngas yield. The CH_4_, CO_2_, and H_2_O conversions at the reforming stage are defined in eqs 19–eq 21. Carbon deposition may occur at the reforming and fuel stages with the deposited carbon released in the forms of CO and CO_2_ at the oxidation stage. It is desired to have minimal carbon deposition to produce syngas with high purity and achieve high CO_2_ capture efficiency. The carbon deposition at the reforming stage is quantified in eq 22. Carbon deposition affects CO selectivity (eq 23). H_2_O production through the RWGS reaction affects H_2_ selectivity (eq 24), while both carbon and H_2_O productions affect the overall syngas selectivity (eq 25). The concentrations and purity of the syngas in the outlet gas stream are affected by the mixing of the carbon, H_2_O, and the unconverted reactants in the reforming stage. Thus, syngas yield quantifies this mixing/dilution effect in eq 26 (Table 2).

**Table 2:** List of Performance Indicators

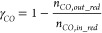 17
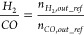 18
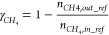 19
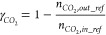 20
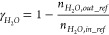 21
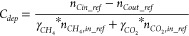 22
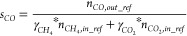 23
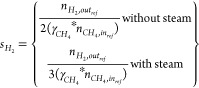 24
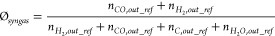 25
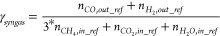 26

### 2.3 Experimental Results and Discussion

The first demonstration of the GSDR concept investigated the effect of the CO_2_:CH_4_ ratio between 1 and 3 which achieved a very low H_2_/CO molar ratio that requires optimization before applying to GTL processes.^11^ In this study, the GSDR process performance is mapped out for GTL integration by further tuning the CO_2_:CH_4_ ratio, investigating the effect of steam addition to the reforming stage, and the effect of the pressurized operation. Except for the pressurized case, CO was used as fuel in the reduction stage to show the possibility of utilizing GTL off-gases where CO forms the largest share in the gas mixture. From the previous study, reducing the degree of this Ni-based oxygen carrier reduction impacts positively the process in terms of improved fuel conversion and reduced carbon deposition.^11^ Therefore, a 50% degree of oxygen carrier reduction was maintained at the reduction stage in this campaign. A control experiment was conducted where the reducing gas was passed through the same amount of solid until no gas conversion was achieved. The total time taken to achieve no gas conversion (100% oxygen carrier utilization) is noted. This time is adjusted to achieve other degrees of reduction; for example, 50% OC utilization is achieved by running the reduction just half of the time that it took for 100% OC utilization. At the reforming stage, CH_4_ and CO_2_/H_2_O were cofed in the presence of metallic Ni (catalyst) for different reactions (Reaction 7–13) to produce syngas (CO + H_2_). The oxidation stage was kept sufficiently long to ensure complete gasification/combustion of any deposited carbon and to fully oxidize the oxygen carrier before starting a new cycle. As mentioned earlier, this demonstration was not authothermal unlike the previous study;^11^ instead, heat was supplied so the reactor temperature drops below 800 °C. This choice is to some extent valid given that heat losses in an industrial GSDR scale will be substantially small; besides, heat integration between the incoming and outgoing flue gases would be used to substantially reduce the temperature variation in the cycle.

#### 2.3.1 GSDR Behavior

**Figure 4:**
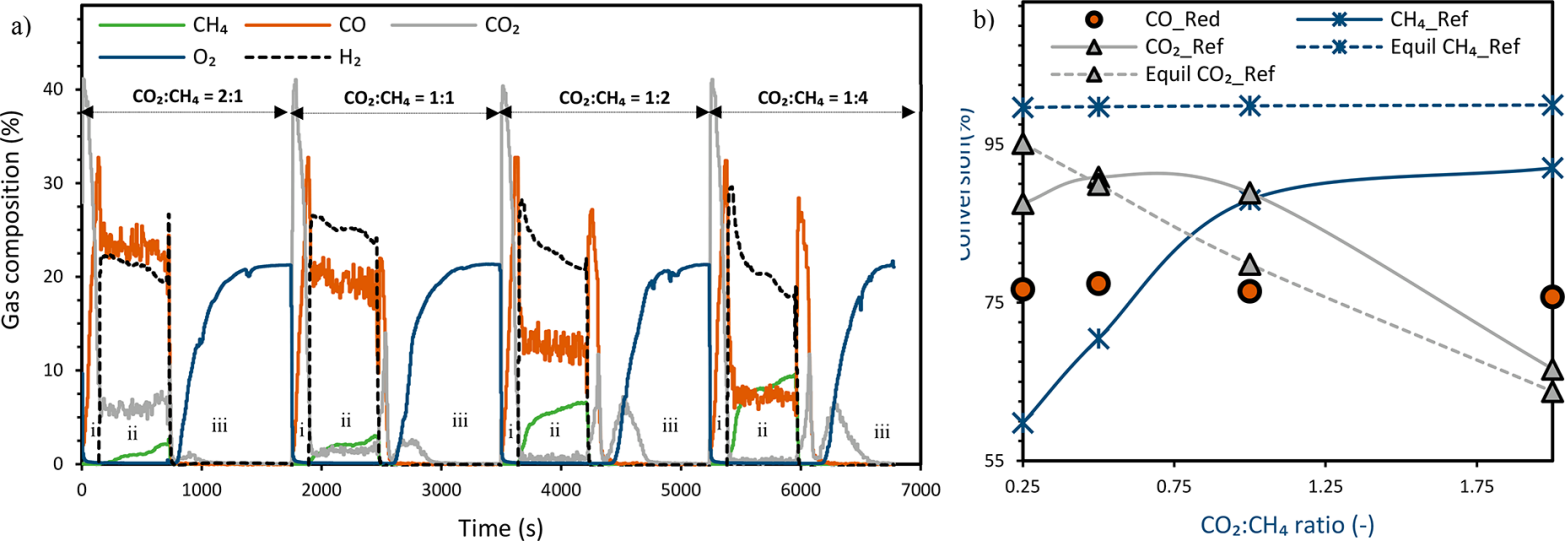
(a) Transient gas composition at the reactor outlet. (b) Gas conversion at different CO_2_:CH_4_ molar ratios at 850 °C and 1 bar. The gas flow rate is as follows: (i) CO 12.8 NLPM at the reduction stage, (ii) CH_4_ 3.2 NLPM, CO_2_ 0.8–6.4 NLPM at the reforming stage, and (iii) air 10 NLPM at the oxidation stage. Note: A known amount of N_2_ was added both in the reduction and reforming stages to quantify the amount of each species leaving the reactor.

Typical behaviors of the GSDR cycle at different CO_2_:CH_4_ molar ratios from 0.25 to 2 are shown through the experimentally measured transient gas composition (Figure 4a). The gas composition at the reduction stage shows that the transient CO conversion is similar for all the cases indicating that the same degree of reduction of the oxygen carrier was achieved before the start of the reforming stage (Figure 4a). The relatively high conversion of CO produces high purity CO_2_ at this stage which can be captured directly without further purification. CO conversion decreases toward the end of the reduction stage possibly due to an increase in carbon deposition. The produced CO_2_ + H_2_O (if CH_4_ is used as fuel) in the reduction stage could be sent to the reforming stage to improve process efficiency and reduce cost.

**Figure 5:**
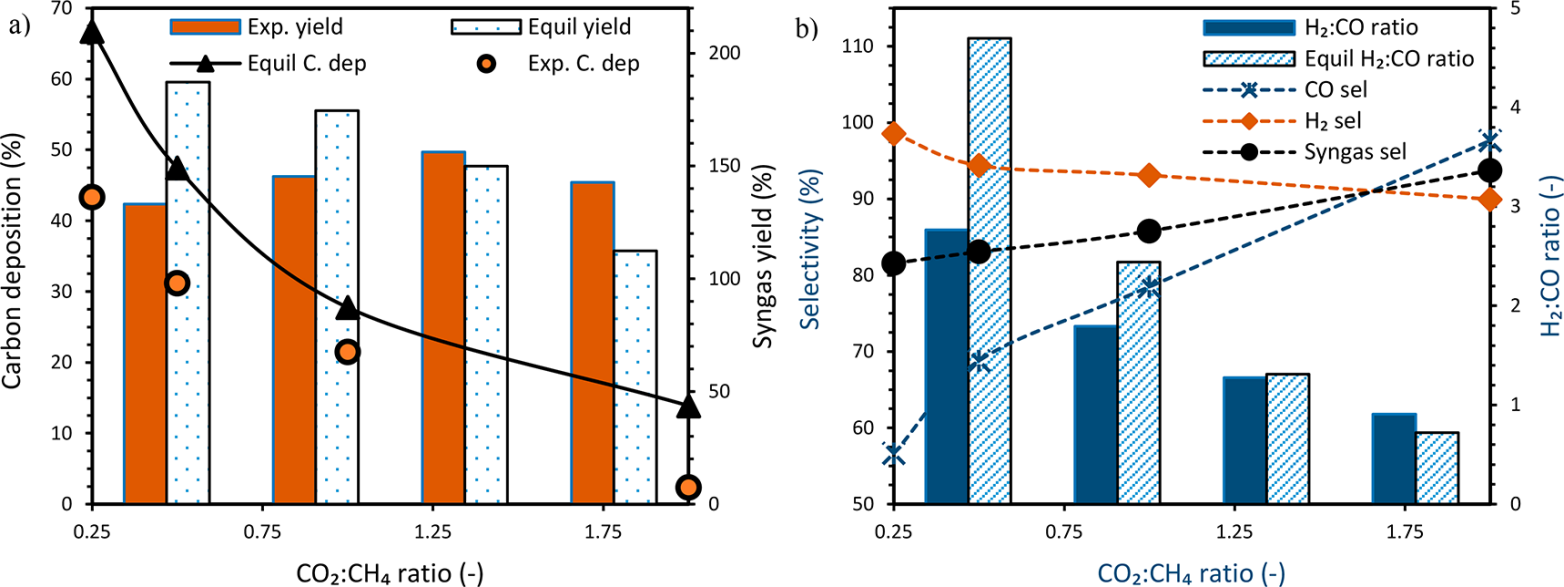
(a) Change of syngas yield and carbon deposition. (b) Change of H_2_ selectivity, CO selectivity, and syngas quality (H_2_/CO molar ratio) with CO_2_:CH_4_ molar ratio in the reforming stage at 850 °C and 1 bar. The gas flow rate is as follows: (i) CO 12.8 NLPM at the reduction stage, (ii) CH_4_ 3.2 NLPM, CO_2_ 0.8–6.4 NLPM at the reforming stage, and (iii) air 10 NLPM at the oxidation stage. Note: A known amount of N_2_ was fed in the reduction and reforming stages to quantify the amount of each species leaving the reactor.

At the reforming stage, it was observed in all cases that CH_4_ conversion decreased throughout the stage (Figure 4a). Interestingly, the CH_4_ slippage escalates in the last two-thirds of the reforming stage with an increased magnitude as the CO_2_:CH_4_ ratio decreases. This behavior is correlated to the extent of carbon deposition that increases rapidly as the CO_2_:CH_4_ ratio decreases (carbon deposition is detected in the oxidation stage shown in Figure 4a in the form of the released CO_2_ due to the oxidation of the deposited carbon using the feed air). Carbon deposition likely becomes more pronounced starting from the last two-thirds of the stage reducing active site availability and thus triggering the escalation in CH_4_ slippage. Nevertheless, Figure 4b shows that the average CH_4_ conversion remains between 60% and 92% for the range of the CO_2_:CH_4_ ratio covered in the study (0.25–2) which is acceptable as the unconverted fuel could be recycled in the cycle for reduction of the oxygen carrier. CO_2_ conversion remains steady across the reforming stage, while the H_2_ fraction decreases (Figure 4a). This could be explained by the fact that the reverse water gas shift (RWGS) (Reaction 9) enhances the conversion of CO_2_ and H_2_ to produce CO and H_2_O despite the decrease in CH_4_ conversion, in agreement with the findings in previous studies.^35,36^ However, CO_2_ conversion decreases with an increase in the CO_2_:CH_4_ ratio driven by the excess CO_2_ in the system (Figure 4b). Nonetheless, the achieved gas conversions remain higher than the result reported in a previous study with a similar impregnated Ni/Al_2_O_3_ catalyst in a fluidized bed with 10 wt % active content^37^ (likely due to higher Ni content) and lower than the conversion from the aerogel Ni/Al_2_O_3_ catalyst^38^ (likely due to the difference in surface area as a result of the production method). CH_4_ conversion was lower than the equilibrium prediction, while the CO_2_ conversion was higher (Figure 4b) confirming the finding in our previous study^11^ and suggesting that kinetics of the different involved mechanisms have higher influences on the process performances. Substantial carbon deposition occurred at a lower CO_2_:CH_4_ ratio but remains below equilibrium predictions (Figure 5a).

Carbon deposition was found to decrease significantly with the increase in the CO_2_:CH_4_ ratio (Figure 5a). Previous studies have confirmed that the first intrinsic step of the dry reforming reaction is methane decomposition to produce H_2_ and carbon (deposit) followed by gasification of the deposited carbon to produce CO.^37,39^ This mechanism explains the observed results given that carbon deposition reduces with the increase in the partial pressure of the oxidant (higher O/C ratio) which is in agreement with previous findings.^28^ With the decrease in carbon deposition, CO selectivity increases, but H_2_ selectivity declines (Figure 5b) as attributed earlier to the RWGS reaction. The high syngas yield achieved is due to the high CO_2_ conversion that exceeded equilibrium prediction. The resulting syngas (H_2_/CO) molar ratio follows a similar trend with carbon deposition confirming its high dependency to the extent of carbon deposition. The release of CO_2_/CO at the air stage (Figure 4a) is due to the combustion/gasification of the deposited carbon thus adversely affecting the CO_2_ capture efficiency and CO_2_ utilization given that the produced gases are vented to the atmosphere with the depleted air. Yet, minimizing carbon deposition in the reduction and reforming stages is crucial for maximizing the environmental and economic impacts of the GSDR process.

In general, varying the CO_2_:CH_4_ ratio from 0.25 to 2 could produce syngas with optimal quality (1< H_2_/CO < 3) and up to 90% syngas purity suitable for GTL processes. However, with this performance, carbon deposition and excess of unconverted CO_2_ from the reforming remain two major challenges to solve for unlocking the expected environmental and economic benefits of GSDR–GTL integration. Carbon deposition does not only have a negative impact on gas conversion and H_2_/CO ratio for GTL applications but also affects CO_2_ utilization negatively. For example, the measured carbon deposition at a CO_2_:CH_4_ molar ratio of 0.25 was ∼43%, implying that the captured CO_2_ for GSDR utilization was barely 57% without accounting for the additional carbon slippage that occurs between the stages. On the other side, the CO_2_ capture efficiency increases to ∼97% at a CO_2_:CH_4_ molar ratio of 2 due to the low carbon deposition of 3%, but over 35% of fed CO_2_ was unconverted and requires implementing a separation step to avoid substantial dilution of the downstream process that may negatively affect its performance. Nonetheless, the major advantage of varying the CO_2_:CH_4_ molar ratio in the gas feed is that it creates flexibility in the process performance and syngas quality for different GTL applications. Having an excess of CO_2_ in the feed results in high methane conversion, high CO_2_ utilization, and reduced carbon deposition but with low syngas purity (high dilution with unconverted CO_2_) and a H_2_/CO ratio < 1. On the other hand, low CO_2_ feed achieves higher purity syngas and a H_2_/CO ratio > 2 but is associated with low methane conversion and high carbon deposition. Further optimization of the GSDR process is therefore needed to minimize the impact of the aforementioned challenges.

#### 2.3.2 Effect of Steam Addition

Dry methane reforming utilizes CO_2_ as feedstock thus offsetting the increasing GHG emission and yields a stoichiometric syngas H_2_/CO molar ratio of 1 which is too low and requires to be tuned up for GTL processes. Steam methane reforming, on the other hand, is slightly less energy intensive but produces a stoichiometric H_2_/CO ratio of 3, which is too high and requires to be tuned down for the downstream GTL processes. Furthermore, dry reforming has a higher tendency toward carbon deposition than steam methane reforming, in agreement with a previous study^40^ which found that steam tends to reduce carbon deposition because of the formation of surface hydroxyl species which inhibits methane decomposition.^41^ To leverage the advantages of these two reactions, it is logical to cofeed H_2_O, CO_2_, and CH_4_ in the reforming stage of the GSDR process to combine the effect of steam reforming, dry reforming, and partial oxidation of methane (with the lattice oxygen of the oxygen carrier) to produce syngas with the desired H_2_/CO ratio for GTL processes with less energy intensity. This approach aligns with the proposed GSDR–GTL integration where GTL off-gases contain H_2_ and CO (in addition to unconverted CH_4_) with different extents depending on the GTL process that will produce H_2_O and CO_2_ in the reduction stage and which will be directed to the reforming stage to produce syngas.

**Figure 6:**
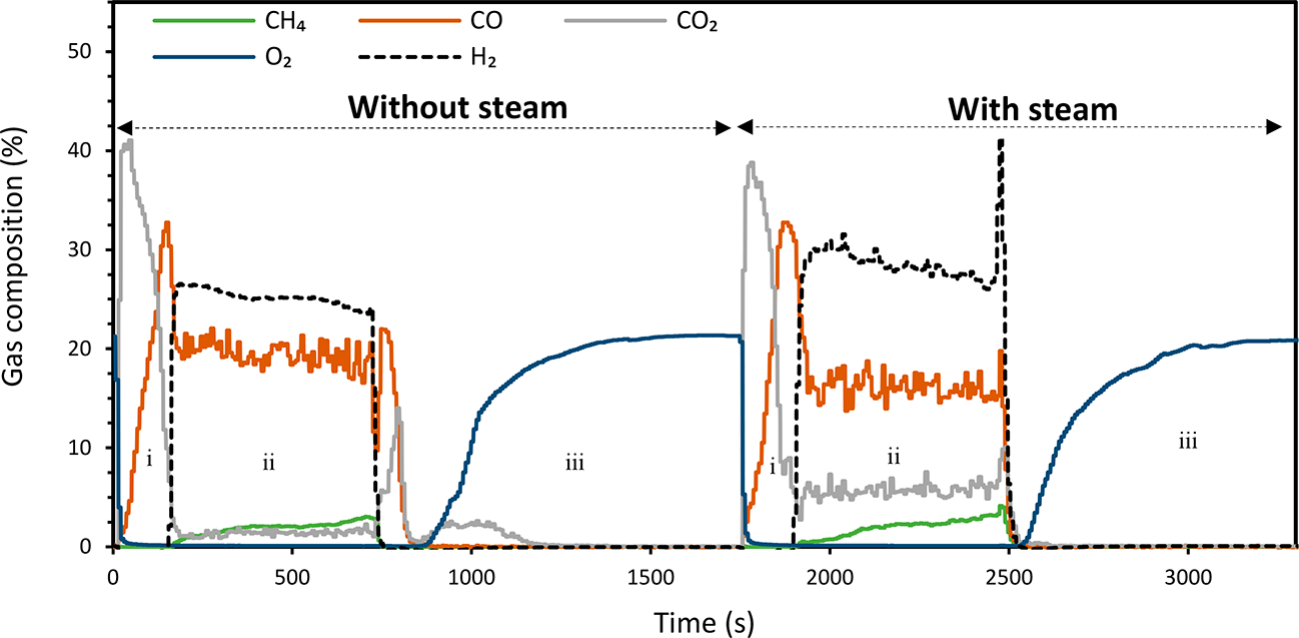
Transient gas composition showing GSDR behavior with and without steam at CO_2_:CH_4_ molar ∼ 1, 850 °C, and 1 bar. Gas flow rate as follows: (i) CO 12.8 NLPM at the reduction stage, (ii) CH_4_ 3.2 NLPM, CO_2_ 3.2 NLPM (H_2_O 3 NLPM for the case with steam) at the reforming, (iii) air 10 NLPM at the oxidation stage. Note: A known amount of N_2_ was fed in the reduction and reforming stages to quantify the amount of each species leaving the reactor.

To demonstrate the effect of steam, three cases (with and without steam) were completed by varying CO_2_:CH_4_ from 0.25 to 1. The range of CO_2_:CH_4_ was chosen where significant carbon deposition was observed. Experiments were completed at atmospheric conditions, 850 °C, and a constant H_2_O/CO_2_ molar ratio of 1 when H_2_O was added. The process behavior could be explained using the transient gas composition at the reactor outlet (Figure 6). As expected, steam addition improved the syngas (H_2_/CO) molar ratio and removed carbon deposition since no CO_2_/CO was produced in the oxidation stage.

**Figure 7:**
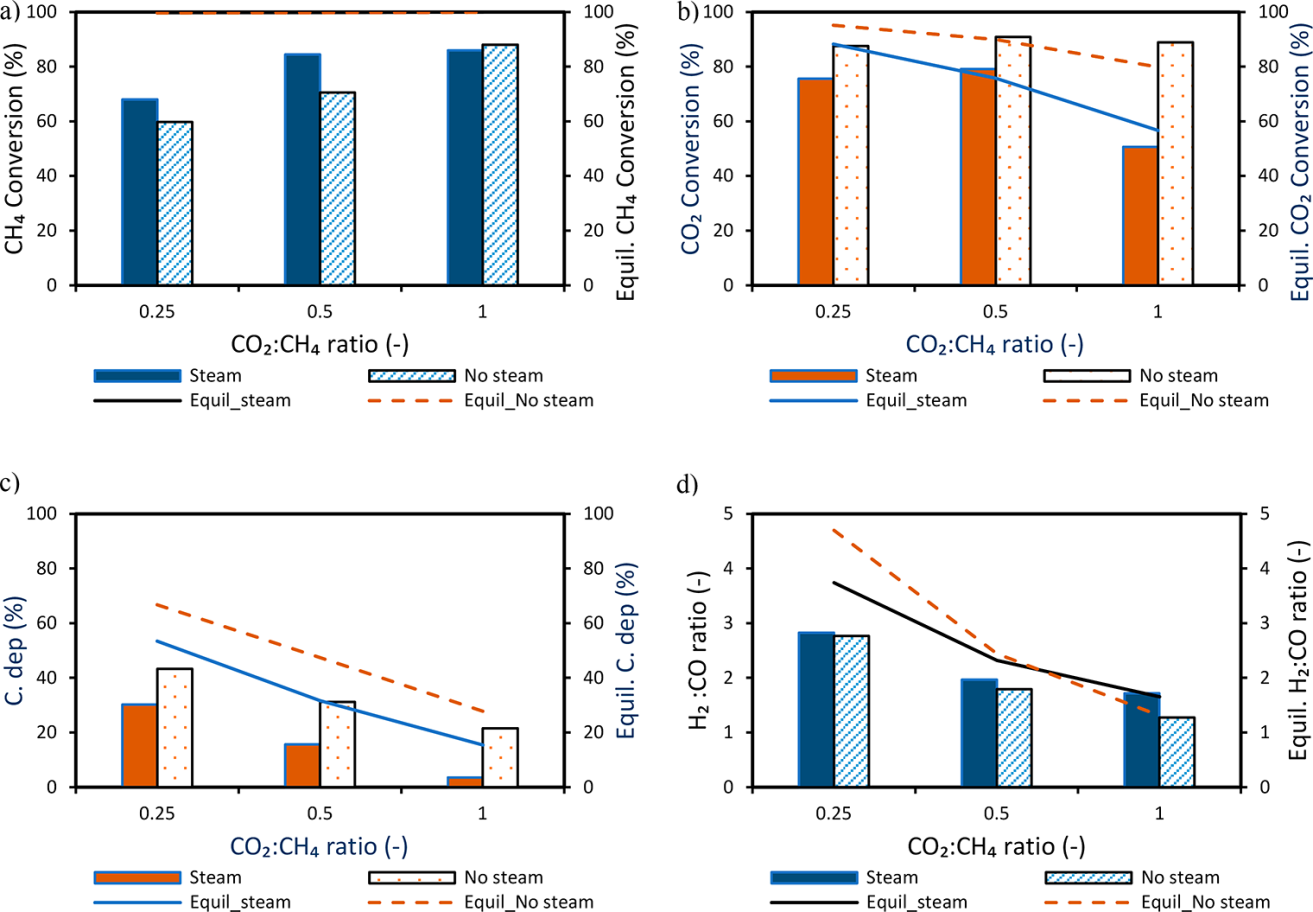
Effect of steam at different CO_2_:CH_4_ molar ratios while maintaining a CO_2_:H_2_O molar ratio of 1: (a) CH_4_ conversion, (b) CO_2_ conversion, (c) carbon deposition, and (d) H_2_:CO molar ratio. Experiments were completed at 850 °C and 1 bar. The gas flow rate is as follows: (i) CO 12.8 NLPM at the reduction stage, (ii) CH_4_ 3.2 NLPM, CO_2_ 0.8–3.2 NLPM, H_2_O 0.75–3 NLPM at the reforming stage, and (iii) air 10 NLPM at the oxidation stage. Note: A known amount of N_2_ was fed in the reduction and reforming stages to quantify the amount of each species leaving the reactor.

**Figure 8:**
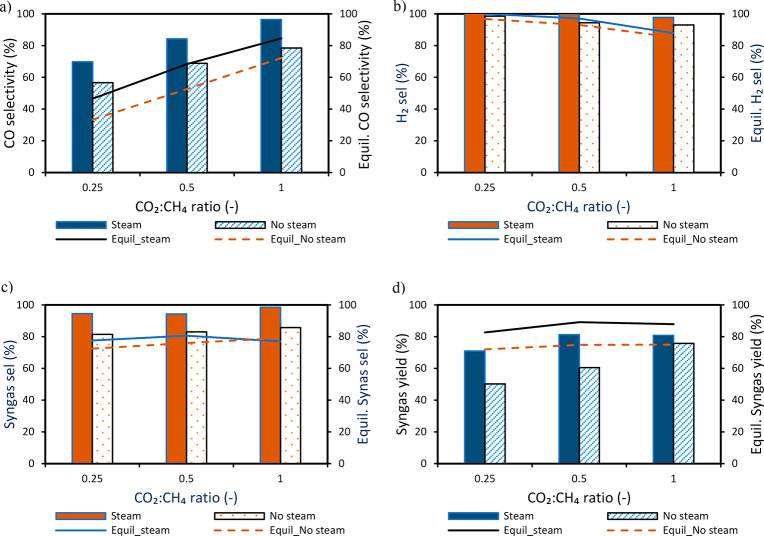
Effect of steam at different CO_2_:CH_4_ molar ratios while maintaining a CO_2_:H_2_O molar ratio of 1: (a) CO selectivity, (b) H_2_ selectivity, (c) overall syngas selectivity, and (d) syngas yield. Experiments were completed at 850 °C and 1 bar. The gas flow rate is as follows: (i) CO 12.8 NLPM at the reduction stage, (ii) CH_4_ 3.2 NLPM, CO_2_ 0.8–3.2 NLPM, H_2_O 0.75–3 NLPM at the reforming stage, and (iii) air 10 NLPM at the oxidation stage. Note: A known amount of N_2_ was fed in the reduction and reforming stages to quantify the amount of each species leaving the reactor.

The effect of steam addition (in the reforming stage) on the different key performance indicators has been illustrated (Figures 7 and 8, respectively). It was observed that steam addition at different CO_2_:CH_4_ molar ratios improved CH_4_ conversion but had a negative effect on CO_2_ conversion (Figure 7b). The increase in CH_4_ conversion (Figure 8a) could be attributed to the higher extent of reforming and gasification reactions since steam is a better gasifying agent than CO_2_ due to its lower dissociation energy as opposed to CO_2_. This behavior conforms with the equilibrium prediction. The expected benefit of steam addition on carbon deposition was also demonstrated, as a reduction in carbon deposition below 3% was achieved with a feed composed of H_2_O:CO_2_:CH_4_ = 1:1:1 molar ratios (Figure 7c). The reduction in carbon deposition was possible due to the increased O/C and H/C ratios in the feed gas suitable for the gasification of carbon.

It was also observed that the syngas (H_2_:CO) molar ratio follows the same trend as carbon deposition (Figure 7d) showing that carbon deposition has a major effect on the syngas quality (H_2_:CO molar ratio). Consequently, CO selectivity was positively affected (Figure 8a) following the improved gasification reaction with steam (Reaction 13). Also H_2_O addition reduced CO_2_ conversion (Figure 7b) counteracting the RWGS reaction (Reaction 9), thus positively affecting H_2_ selectivity (Figure 8b). The overall syngas selectivity (Figure 8c) and yield (Figure 8d) improve with steam addition, while the decline in syngas yield shown when the CO_2_:CH_4_ ratio increased to 1 is due to the excess CO_2_ in the product gas as a result of the decline in CO_2_ conversion.

In summary, steam addition resulted in improvement in syngas quality (H_2_/CO molar ratios close to 2 could be achieved at 0.5 < CO_2_/CH_4_ < 1 with H_2_O feed equal to CO_2_) providing an additional variable to further control this important parameter when considering efficient integration with GTL processes. It also minimized carbon deposition thus improving the ability of the GSDR process to efficiently capture carbon for ultimate utilization in GTL. It should be noted that the needed steam could be directly sourced from the reduction stage if methane is used as a reducing agent or if the GTL off-gases contain unconverted hydrogen. When GSDR is operated autothermally, for converting one mole of CH_4_ through dry reforming (Reaction 7), 247 kJ of heat is required to be supplied which is equivalent to the heat generated from the combustion of ∼0.3 mol of CH_4_ (the standard heat of combustion of CH_4_ is taken as 802 kJ/mol). This will produce 0.3 mol of CO_2_ and 0.6 mol of H_2_O to be supplied to the reforming stage to bring the benefits of minimized carbon deposition and adequate syngas quality. This estimation was made assuming adiabatic conditions, and no sensible heat is needed to heat the feed gases from room temperature to the reaction operating temperature which is valid if proper heat integration is applied.

#### 2.3.3 Effect of pressure

GTL processes operate at elevated pressures; therefore, it is necessary to also operate the syngas production step at high pressures to make the proposed integration to the downstream GTL processes efficient. From a process optimization point of view, high-pressure operations would also increase process capacity, reduce equipment sizes, and cost.^42^ Therefore, understanding the effect of operating pressure on the GSDR syngas generation step is essential to the design of the overall system.

**Figure 9:**
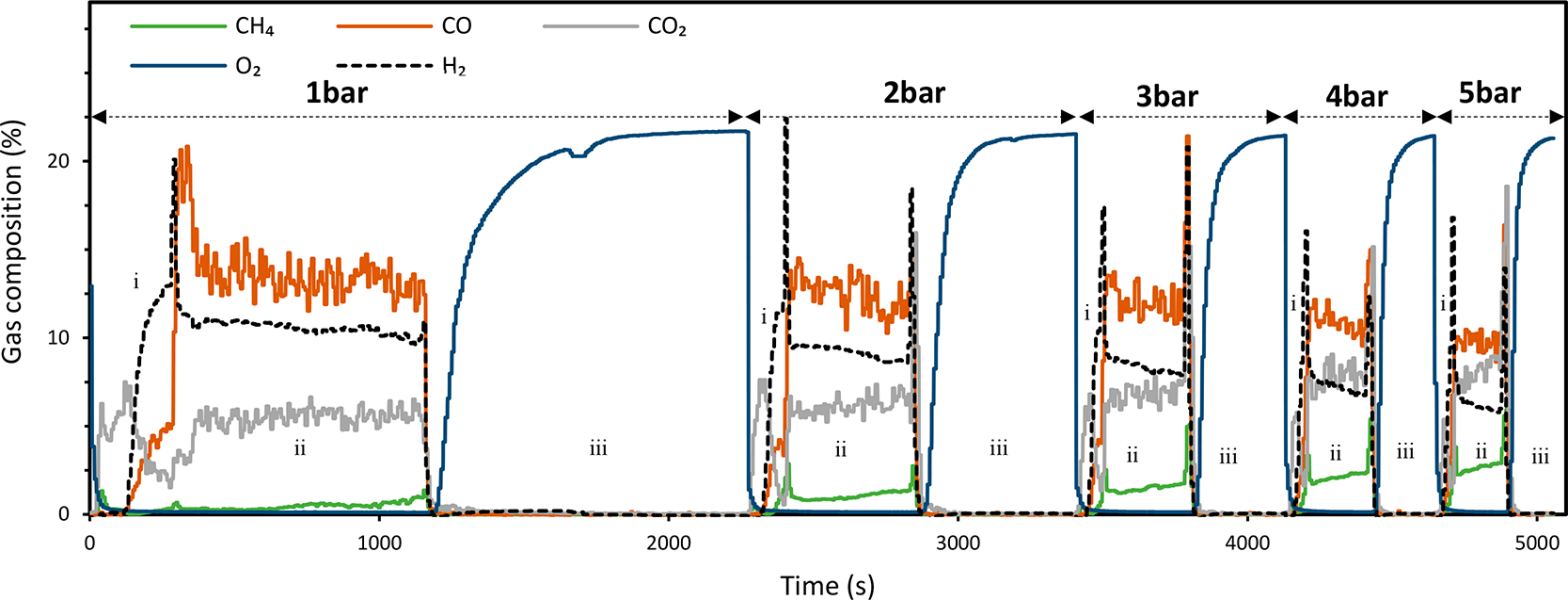
Transient gas composition for different pressures (1–5 bar) at a CO_2_:CH_4_ molar ratio of 2 and 850 °C. The gas flow rate is as follows: (i) CH_4_ 1–5 NLPM at the reduction stage, (ii) CH_4_ 1–5 NLPM, CO_2_ 2–10 NLPM at the reforming stage, and (iii) air 10–50 NLPM at the oxidation stage. Note: A known amount of N_2_ was fed in the reduction and reforming stages to quantify the amount of each species leaving the reactor.

**Figure 10:**
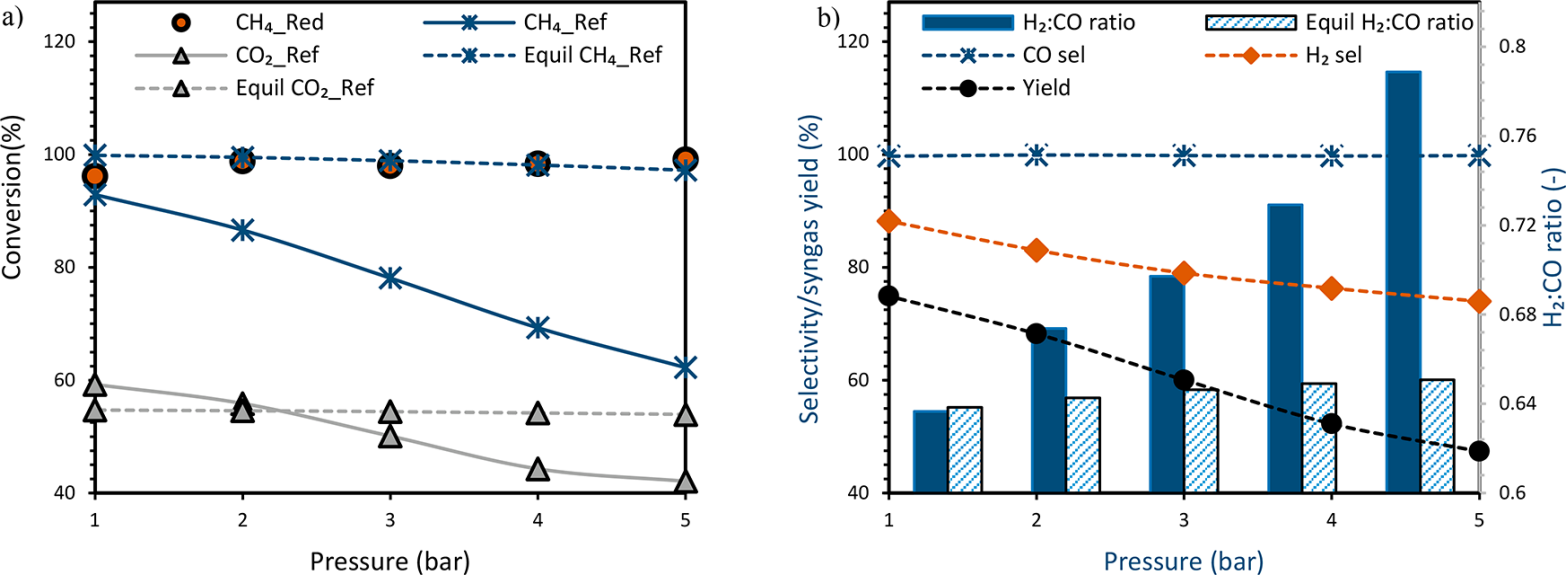
(a) Variation of gas conversion and (b) variation of selectivity, yield, and syngas quality (H_2_/CO molar ratio) with pressure at a CO_2_:CH_4_ molar ratio of 2 and 850 °C. The gas flow rate is as follows: (i) CH_4_ 1–5 NLPM at the reduction stage, (ii) CH_4_ 1–5 NLPM, CO_2_ 2–10 NLPM at the reforming stage, and (iii) air 10–50 NLPM at the oxidation stage. Note: A known amount of N_2_ was fed in the reduction and reforming stages to quantify the amount of each species leaving the reactor.

The effect of pressure on the GSDR performance was investigated at a CO_2_/CH_4_ ratio of 2 (this value was chosen to reduce carbon deposition as illustrated in Section 2.3.1). Pure CH_4_ was used in the reduction stage, while CO_2_ was added only in the reforming stage. The feed rates to each stage were increased proportionally to the pressure to maintain the residence time constant. Figure 9 shows the transient gas composition at the reactor outlet of the GSDR cycle for the different operating pressures investigated in this study. The result shows that CH_4_ conversion was high in the reduction stage, although two different substage behaviors were observed. The first substage is associated with high selectivity to combustion (CO_2_ and H_2_O), while in the second substage, CH_4_ converts mainly to syngas (H_2_ + CO). These behaviors were also observed in previous studies,^11^ where the behavior at the first substage is attributed to the easy access to oxygen at the start of the reduction stage leading to full combustion of methane. The partial oxidation of methane to syngas shown at the second substage starts once enough metallic Ni sites become available to catalyze the reforming reactions. Nevertheless, the overall CH_4_ conversion in the reduction stage was high and insensitive to the pressure (Figure 10a) implying that the oxygen carrier achieves relatively the same reduction level before starting the dry reforming stage. CH_4_ and CO_2_ conversion in the reforming stage were negatively affected by the pressure showing increased slippage (Figure 9) and leading to reduced syngas yield as the pressure is increased (Figure 10b). This agrees with thermodynamics but with a larger extent for the experimental results (Figure 10a). The result also indicates that elevated pressure slows the kinetics of the dry reforming reaction with this specific oxygen carrier, a challenge that could be compensated for by operating at higher temperatures as suggested in previous studies^7,43^ or by selecting a better oxygen carrier/catalyst to improve the kinetics of the process.^7^

CO selectivity was improved slightly with an increase in pressure due to the positive effect of pressure in reducing carbon deposition (carbon deposition completely eliminated at 5 bar). Surprisingly, H_2_ selectivity was negatively affected against equilibrium predictions. This result could be explained by the larger excess of CO_2_ left in the system due to the negative effect of pressure on CO_2_ conversion, which counteracted the adverse effect of pressure on RWGS (Reaction 9) leading to decreased H_2_ production. Consequently, this leads to overall syngas quality (H_2_/CO molar ratio) deterioration as the pressure is increased (Figure 10b).

Despite the small range of pressure covered in this study, it has given a clear idea of how fast the performance of the reforming stage can deteriorate when the pressure is increased. In principle, a drop of up to 30% in methane conversion could be accommodated given that the unconverted fuel will be utilized in the reduction stage needed for heat supply to the GSDR process. However, the current results revealed that with the current oxygen carrier, CH_4_ conversion will drop to 39.36% at 20 bar and to 26.28% at 40 bar, while CO_2_ conversion drops to 27.62% and 19.91% for the two operating pressures, respectively (assuming a fitted logarithmic change of gas conversion with pressure). Obviously, increasing the operating temperature would reduce the impact of pressure on methane conversion, but it is unlikely that thermodynamic conversion could be achieved at economically low temperatures with this oxygen carrier. Extreme operating temperatures would pose new challenges linked to oxygen carrier mechanical stability and involve the need for special expensive alloys to withstand the combined high-pressure high-temperature reactive conditions.

Considering these numbers, finding an oxygen carrier with improved catalytic activity for the dry reforming reaction is necessary for achieving the expected economic and environmental benefits of the GSDR concept for integration with GTL processes. An optimal candidate should achieve near 70% methane conversion (the equilibrium conversion at 850 °C and 20 bar), while the unconverted 30% could be recycled to the reduction stage. Improving the rate of dry reforming reaction will not only enhance the conversion of CO_2_ to syngas but also limit the RWGS reaction, making it possible to achieve an optimal H_2_/CO molar ratio (1–2) suitable for GTL processes.

## 3 GSDR–Methanol Process Integration

**Figure 11:**
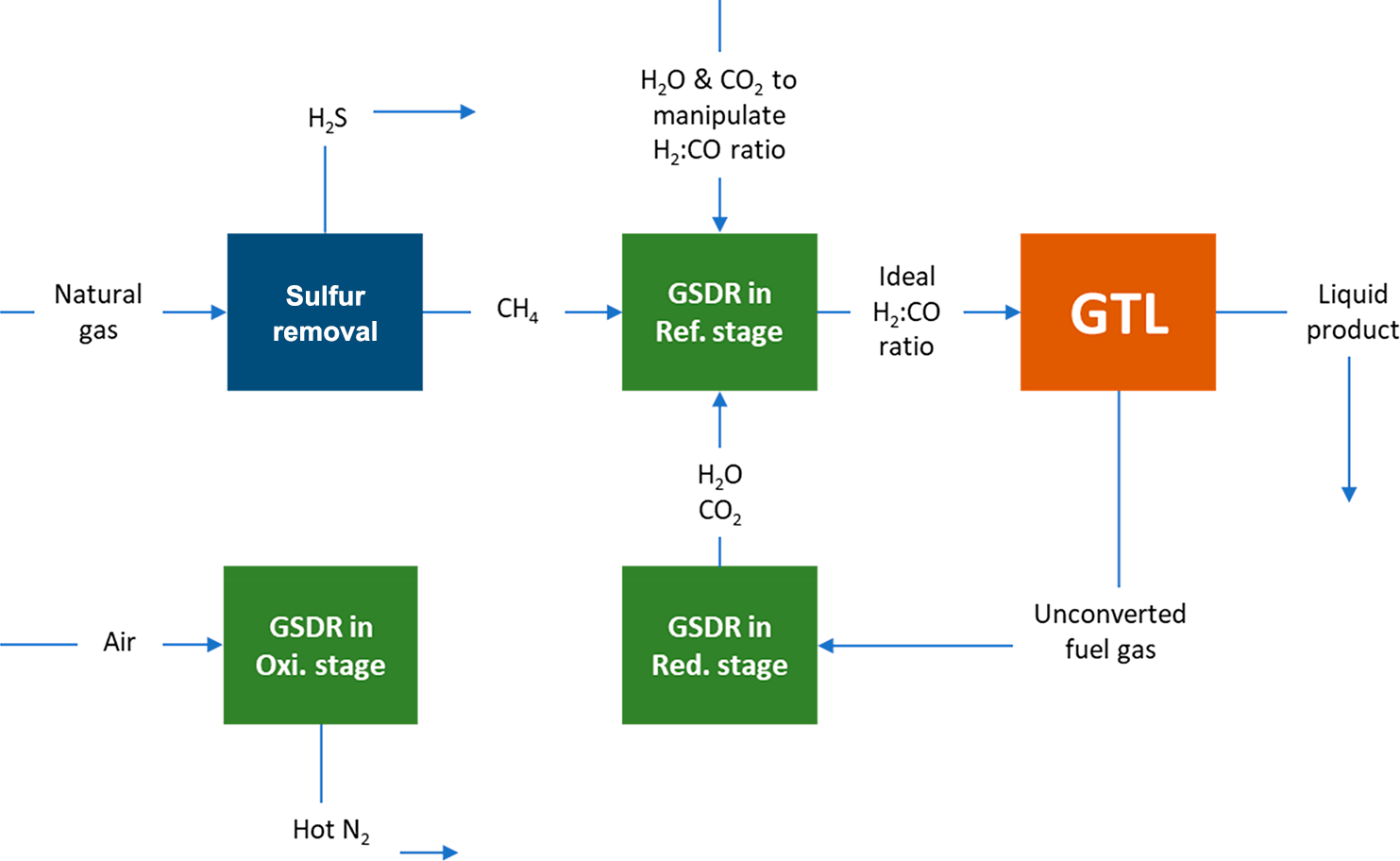
Proposed GSDR–GTL integration.^11^

As explained in the Introduction, the main aim of this study is to explore ways to introduce the GSDR process as an alternative for syngas production for GTL processes (Figure 1). This section, therefore, demonstrates how this can be achieved through process modeling by integrating the GSDR process into a state-of-the-art methanol plant^44^ to supply syngas for Reactions S6 and S7 in the Supporting Information. The process is benchmarked with state-of-the-art autothermal reforming (ATR).^45^ A schematic diagram of the GSDR–MeOH process integration is shown in Figure 11. The GSDR part consists of three stages (reduction, reforming, and oxidation), and the process starts with the reduction stage (Figure 11) where the unconverted gas from a GTL process is utilized (recycled) to reduce NiO to the metallic state (Ni). The product of the reduction reaction (CO_2_ and H_2_O) is sent to the reforming stage where CH_4_, CO_2_, and/steam react in the presence of a Ni/NiO catalyst to produce syngas (CO and H_2_). The outlet gases from this stage, containing mainly syngas with the desired H_2_/CO molar ratio of ∼2, is sent to the methanol production process. The oxidation stage (Figure 2) starts after the reforming stage where pure air is fed to reoxidize the reduced oxygen carrier (Ni) to NiO and generate heat needed for the endothermic reforming reaction.

### 3.1 Process Design

**Figure 12:**
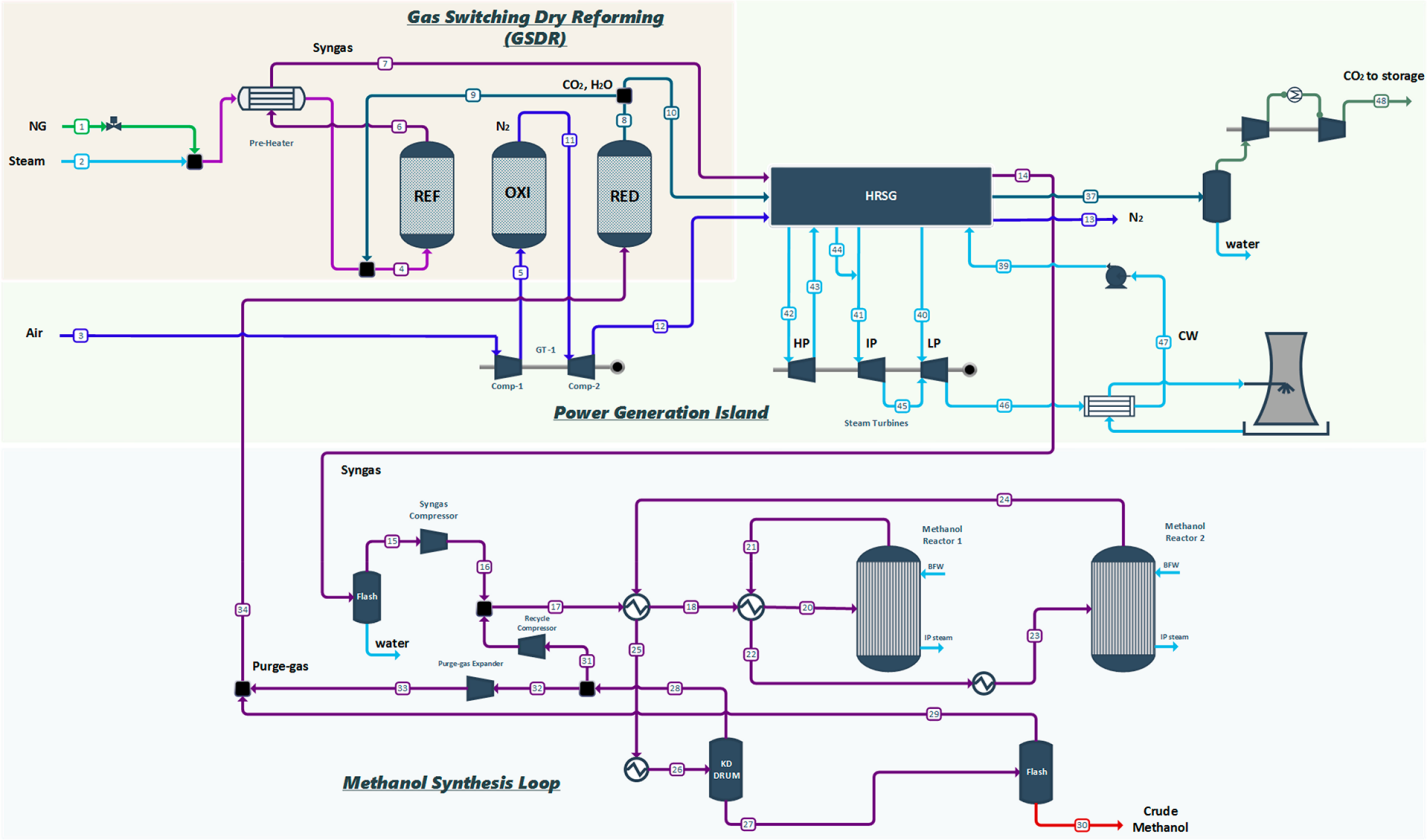
Process flow diagram of GSDR-based methanol production plant.

Figure 12 shows the schematic illustration of the integrated GSDR and the methanol synthesis process. The GSDR process consists of three fluidized bed dynamically operated reactors to represent the three stages—the reforming (REF), oxidation (OXI) and reduction (RED) stages. A nickel-based (30 wt %) oxygen carrier (OC) supported on Al_2_O_3_ was used in the simulation to supply oxygen (in form of lattice oxygen) for the redox reactions. The OC when reduced to the metallic state (Ni) acts as a catalyst for the reforming reactions that occur during the reforming stage (REF) as explained in Section 1. The processes for the methanol synthesis loop, power generation island and ATR are similar to the previous study^44^ and are described in the Supporting Information. However, the process parameter and conditions are different in this study, adapted to ensure optimal integration of the proposed GSDR process in MeOH as shown in Tables A1 and B1, respectively, in the Appendix. It should be noted that the GSDR process has a different working principle than the internally circulating reactor concept that was used in the previous study^44^ imposing new boundary conditions for integration in methanol production. In GSDR, the methanol plant off-gases are fed to the reduction stage, and the products are fed to the reforming stage, while in ICR^44^ the methanol plant off-gases are cofed with methane to the reformer. It has been shown previously that a separate reduction in GSDR improves methane conversion in the reforming step given that methane as the only gas fed reduces the oxygen carrier more to catalyze the reforming reaction.^46^

### 3.2 Process Modeling

The GSDR-MeOH process was modeled in Aspen Plus through guidance from the experimental study about the GSDR operation sequence and performance (assuming the deterioration in the GSDR performance at high pressure could be overcome by using an optimized oxygen carrier). A state-of-the-art methanol process^44^ was chosen with some modifications in the process parameters and conditions to suit the design of the proposed GSDR process, while the ATR system was modeled based on the DOE natural gas to methanol report.^45^ The process modeling and mass/energy balance calculations used for the technical performance evaluations were performed using Aspen Plus V10.0. The thermodynamic properties were evaluated using the Soave–Redlich–Kwong equation of state with the Boston–Mathias alpha function (RKS-BM) for all systems except the steam Rankine cycle where the steam tables (STEAM-TA) were used.

Thermodynamic equilibrium was assumed, and the product compositions were evaluated using the Gibbs energy minimization technique. Table 3 summarizes the general parameters and assumptions used for the process simulations. Figure 12 shows the process flow diagram of the methanol production plant integrated with gas switching reforming (GSDR), while the process flow diagram of the autothermal reforming (ATR) is shown in the Supporting Information. The methanol plant consists of three main parts: (1) the syngas production part through natural gas reforming, (2) the power generation part using a Brayton and Rankine combined cycle system, and (3) the methanol synthesis part. The performances of the ATR and GSDR systems were compared based on the same basis by keeping the natural gas input constant for all the cases at the same methanol production capacity (∼10,000 TPD). The following indicators–equivalent methanol efficiency (η_*MeOH,equ*_), methanol production efficiency (η_*MeOH*_,), and the CO_2_ specific emission (*E*_*CO*_2__) were defined to quantify each process performance as described in a previous study.^44^

**Table 3:** Process Simulation Specifications and Assumptions

Methanol production capacity	∼10,000 TPD, ∼116.5 kg/s		
	
Natural gas	Composition (mass%):		
	CH_4_ = 93.1, C_2_H_6_ = 3.2, C_3_H_8_ = 0.7, C_4_H_10_ = 0.4, CO_2_ = 1.0, N_2_ = 1.6		
	LHV (kJ/kg): 47.454		
	HHV (kJ/kg): 52.581		
	
Thermodynamic property methods	Soave–Redlich–Kwong (RKS-BM) and Steam Tables		
			
Turbomachines modeling parameters	Compressors	η_*is*_ = 0.86	η_*me*_ = 0.99
	Gas turbine	η_*is*_ = 0.86	η_*me*_ = 0.99
	HP steam turbine	η_*is*_ = 0.85	η_*me*_ = 0.99
	IP steam turbine	η_*is*_ = 0.9	η_*me*_ = 0.99
	LP steam turbine	η_*is*_ = 0.75	η_*me*_ = 0.99
	Pumps	η_*is*_ = 0.75	η_*me*_ = 0.95
			
Reactor module type	GSDR (REF, RED, OXI)	RGibbs	
	ATR	RGibbs	
	Methanol synthesis	REquil	
	
Heat recovery steam generation (HRSG) and steam cycle	Three pressure levels (HP/IP/LP): 140/20/5 bar, 560/320/160 °C		
	Condensation pressure = 0.04 bar.		

Methanol reactors	Reactor 1: temperature 246 °C, pressure 50 bar		
	Reactor 2: temperature 220 °C, pressure 50 bar		
	
Air separation unit (ASU)	Oxygen purity: 95% (vol.)		
	Power consumption: 122 MW_el_		

#### 3.2.1 Plant Performance Indicators

Given that the methanol plant produces two different energetic outputs as methanol product and electricity, the technical performances of the GSDR and ATR-based plants were compared based on the equivalent methanol efficiency, which is defined as
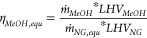
27
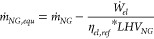
28

The methanol production efficiency η_*MeOH*_ (eq 29) is defined as
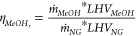
29where η_*MeOH,equ*_ is the equivalent methanol efficiency, *ṁ*_*MeOH*_ the produced methanol (kg/s), *ṁ*_*NG*_ the feed natural gas (kg/s), *LHV*_*i*_ the lower heating value (MJ/kg), *Ẇ*_*el*_ the net power output (MW), *ṁ*_*NG,equ*_ the equivalent natural gas input, and η_*el,ref*_ the reference equivalent natural gas power plant efficiency assumed to be 58.3%.

The CO_2_ specific emission (E_CO2_,g_CO2_/MJ_methanol_) (eq 30) is defined as the amount of CO_2_ emitted in the process per unit mass of methanol produced.
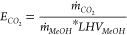
30

### 3.3 Process Performance

**Figure 13:**
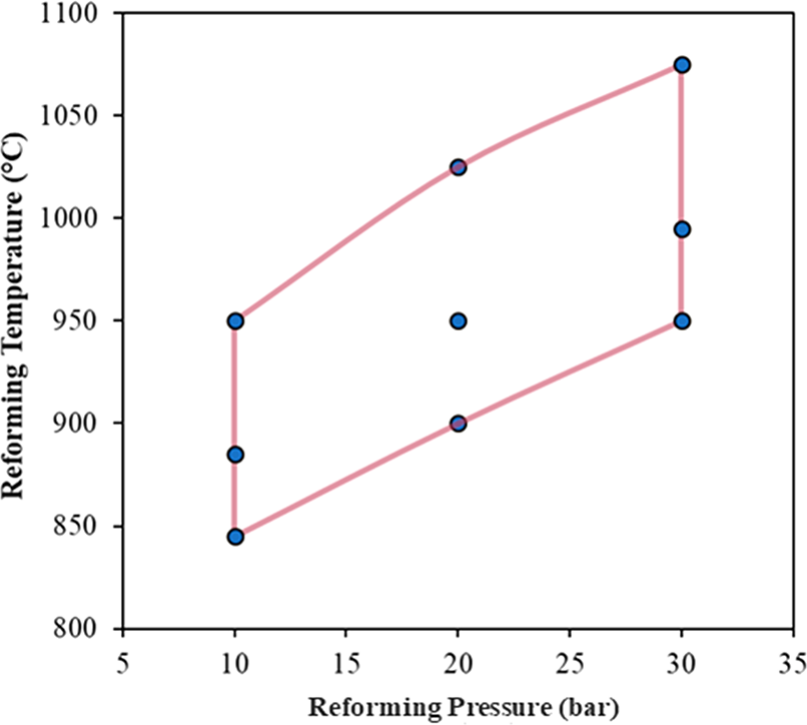
Reforming temperature and pressure conditions of the different simulation points.

**Figure 14:**
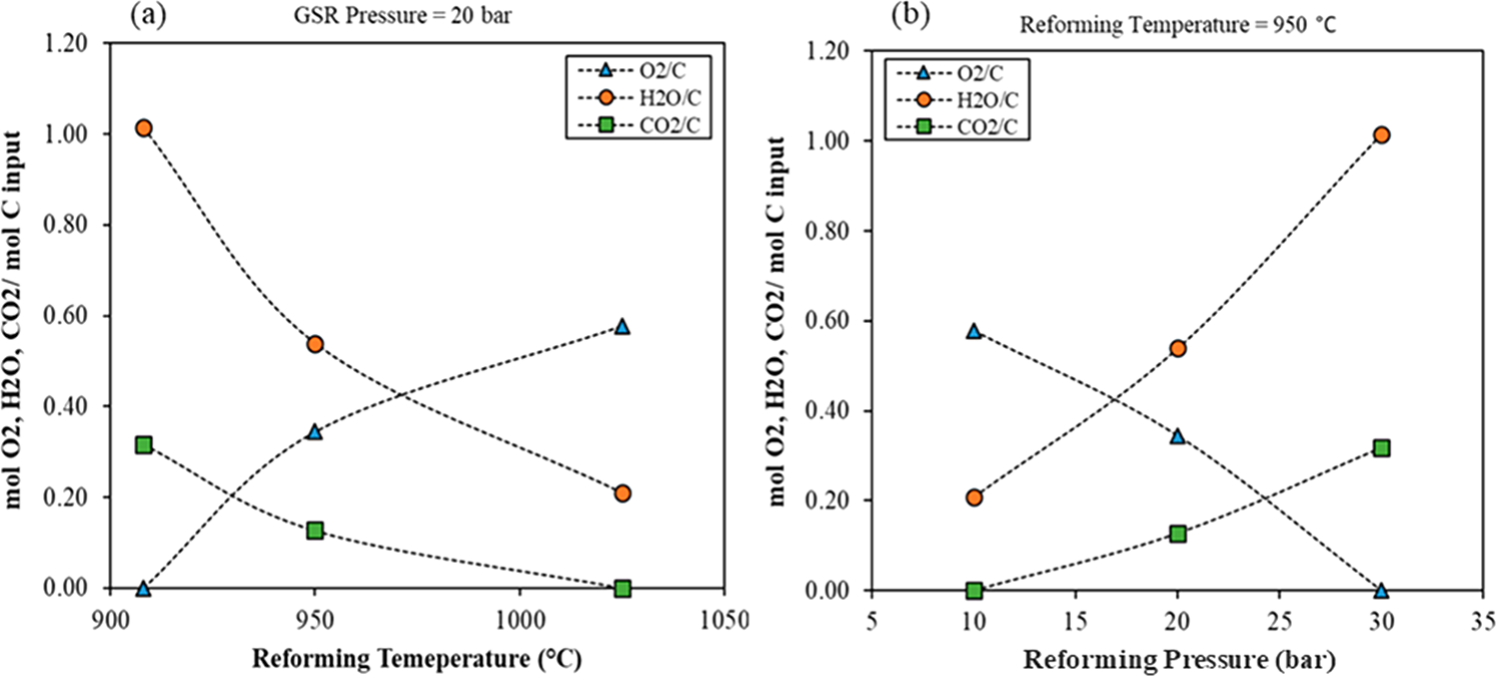
Variation of O_2_/C, H_2_O/C, and CO_2_/C in the feed to the reforming stage for maintaining similar produced syngas composition with (a) reforming temperature at 20 bar and (b) reforming pressure at 950°C.

This section presents the obtained results from the process simulation of the methanol production plant that relies on the proposed GSDR concept as a syngas source. The process performance was investigated by varying the temperature and pressure of the reforming step of the GSDR process from 845–1075 °C and 10–30 bar, respectively, as shown in Figure 13. The higher-pressure cases were operated at higher temperatures to compensate for the drop in methane conversion imposed by equilibrium (Figure 13). In all cases, ideal mixing and equilibrium conversion were assumed, and methanol production was kept constant at 116.5 kg/s. The aim was to find optimum operating conditions and compare the GSDR performance at various temperatures/pressures with the ATR-based methanol plant. Figure 14 shows the feed conditions of the GSDR process in terms of molar fraction (O_2_/C, H_2_O/C, and CO_2_/C) inputs at various pressures and temperatures. At constant temperature, increasing the GSDR pressure will require increasing both H_2_O/C and CO_2_/C inputs while decreasing the O_2_/C input ratio to maintain a similar produced syngas composition (H_2_/CO = 2 and M = 1.7), which yields a fixed methanol production capacity of 116.5 kg/s. M = (H_2_ – CO_2_)/(CO + CO_2_), a feed parameter for an optimal yield of methanol (see Section 1.3 of the Supporting Information). On the contrary, increasing the temperature while keeping the GSDR pressure constant requires decreasing both H_2_O/C and CO_2_/C inputs while increasing the O_2_/C input ratio. The summary of the operation conditions is presented in Table 4, while the mainstream conditions, flow rates, and gas compositions for the GSDR and ATR cases are shown in Tables A1 and B1, respectively.

**Table 4:** GSDR Operating Conditions

GSDR pressure (bar)	10	10	10	20	20	20	30	30	30
NG feed (kg/s)	73.54	73.54	73.54	73.54	73.54	73.54	73.54	73.54	73.54
Temperature, REF (°C)	845	885	950	908	950	1025	950	995	1075
Temperature, OXI (°C)	990	1025	1082	1051	1088	1154	1092	1131	1202
Temperature, RED (°C)	950	1003	1073	1011	1066	1146	1052	1110	1195
O_2_/C input	0.00	0.34	0.58	0.00	0.34	0.58	0.00	0.34	0.58
H_2_O/C input	1.01	0.54	0.21	1.01	0.54	0.21	1.01	0.54	0.21
CO_2_/C input	0.32	0.13	0.00	0.32	0.13	0.00	0.32	0.13	0.00
OC utilization %, RED	100	72	43	100	72	43	100	72	43
Proportion of syngas sent to MeOH plant, %	89	93	96	88	93	96	87	93	96
Synthesis loop flow rate, kg/s	627	603	579	605	645	581	563	614	585
H_2_/CO (Syngas)	2.3	2.1	2.0	2.2	2.1	2.0	2.2	2.1	2.0
M (Syngas)	1.7	1.7	1.7	1.7	1.7	1.7	1.7	1.7	1.7
Methanol production (kg/s)	116.5	116.5	116.5	116.5	116.5	116.5	116.5	116.5	116.5

**Figure 15:**
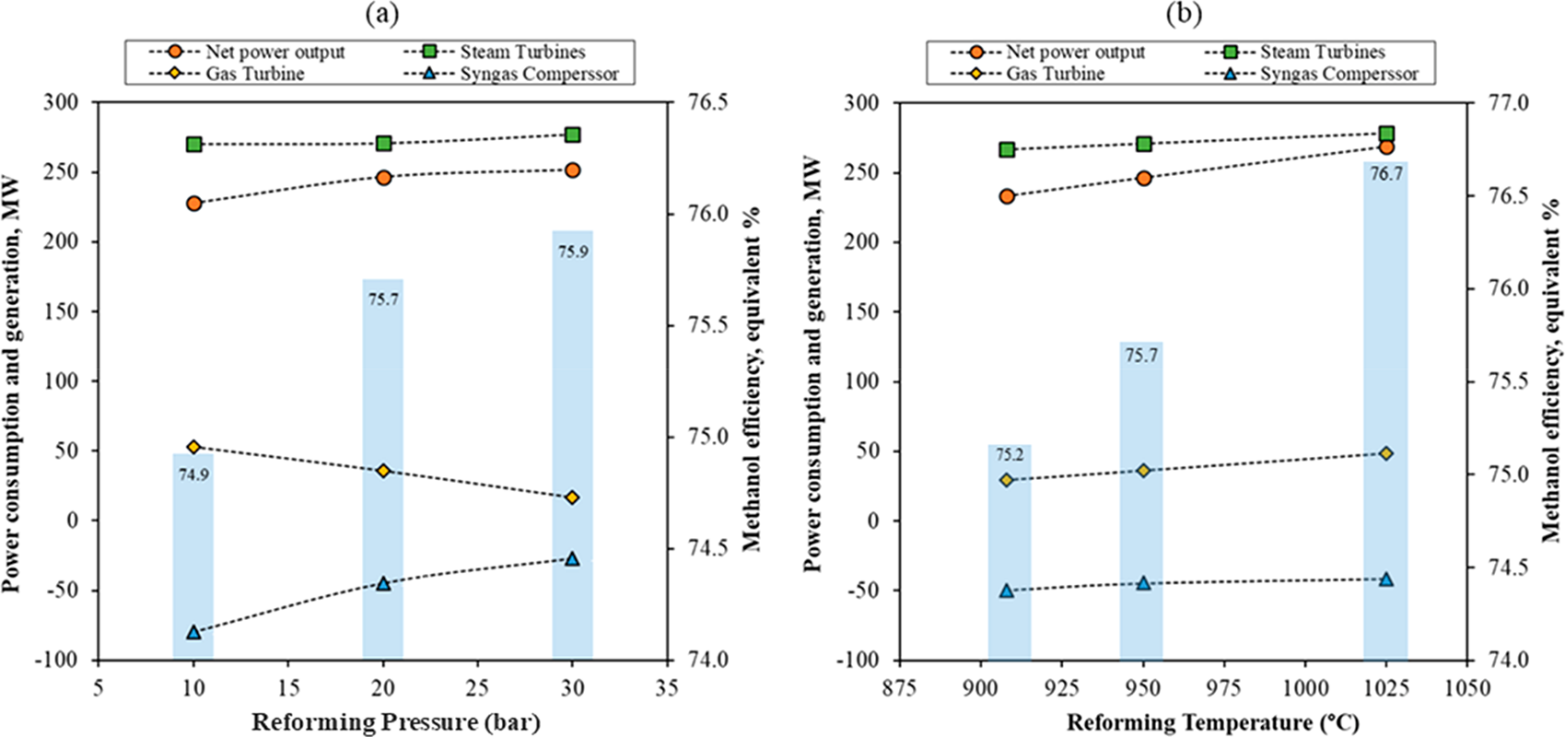
Effect of (a) reforming pressure at 950 °C and (b) reforming temperature at 20 bar on power consumption and methanol efficiency.

Figure 15 shows how the reforming temperature and pressure affect the power consumption and the methanol production efficiency. Trends similar to those found in ref (44) can be observed. By increasing the GSDR pressure (at constant reforming temperature 950 °C) from 10 to 30 bar (Figure 15 a), the overall plant efficiency improves by 1% as the syngas compressor duty (blue tringle) decreases by 66.2% since syngas is delivered at pressures closer to the methanol process. On the other hand, the gross power output from the gas turbine (defined as gas turbine output minus the air compressor duty of the GSDR) decreases by 68.1% by increasing the pressure from 10 to 30 bar due to the higher air compressor duty required to pressurize the GSDR loop. The steam turbine power output was found to be insensitive to the GSDR pressure.

By increasing the reforming temperature from 908 to 1025 °C (at 20 bar), the overall process efficiency improves by 1.5% (Figure 15 b). This is mainly due to the increase in the power output (from the gas and steam turbine) and a slight decrease in syngas compression duty at higher temperatures. The gross power output from the gas turbine increases by 65.3% because of the increase in the oxidation stage temperature that delivers depleted air gas at higher temperature to turbine. The steam turbine power output slightly increases with an increase in temperature because of the hotter syngas stream and hence higher steam generation in the HRSG. Additionally, a lower S/C ratio was used at higher reforming temperature; hence, more steam was utilized for power generation instead of feeding to the reformer. The syngas compressor duty decreases 16.8% due to the decrease in the total volumetric flow rate that the compressor handles since the amount of unconverted CH_4_ decreases due to the improvement in CH_4_ conversion at high reforming temperatures. From thermodynamic calculations, an increase in pressure reduces CH_4_ conversion, the syngas yield of the GSDR process, and the amount of methanol produced. Therefore, it is important to operate within an optimum balance of temperature and pressure to achieve good methanol yield without compromising efficiency.

The technical performance is summarized in Table 5, and it can be shown that the MeOH plant efficiency of the reference ATR case (at 25 bar and 1059 °C) is 73.9%, while GSDR (at 20 bar and 1025 °C) is 76.7%. The overall process efficiency of ATR is lower that GSDR because of the high energy consumption associated with air compression and separation units of the ATR process as against the GSDR where additional process units for air separation are not required. The additional attractiveness of GSDR is that CO_2_ is rather utilized in the process to produce syngas as opposed to the reference ATR case where part of the O_2_ supplied is used to directly combust the fuel (for heat generation) and at the same time reform/partially to oxidize CH_4_ to produce syngas with associated CO_2_ emissions. The specific CO_2_ emission of the reference ATR (at 25 bar and 1059 °C) is 14.7 gCO_2_ for every MJ of methanol produced.

**Table 5:** Technical Performance of GSDR-Based Methanol Plant at Various Pressures and Temperatures and ATR-Based Plant

	GSDR									ATR
NG feed (kg/s)	73.54	73.54	73.54	73.54	73.54	73.54	73.54	73.54	73.54	73.54
Methanol production (kg/s)	116.5	116.5	116.5	116.5	116.5	116.5	116.5	116.5	116.5	116.5
GSR/ATR pressure (bar)	10	10	10	20	20	20	30	30	30	25
REF/ATR temperature	845	885	950	908	950	1025	950	995	1075	1059
										
**Power consumption** (MW)										
Air separation unit, ASU	0	0	0	0	0	0	0	0	0	–122
Air compressor 1 (Comp-1)	–144.5	–144.4	–144.5	–203.5	–203.4	–203.3	–244.4	–243.8	–243.5	0
Air compressor 2 (Comp-2)	0	0	0	0	0	0	0	0	0	–100.7
CO_2_ compressor	–13.8	–13.8	–13.3	–12.9	–12.9	–12.7	–12.8	–12.8	–12.7	0
Syngas compressor	–95.6	–86.1	–79.3	–49.5	–44.5	–41.2	–26.8	–24.1	–22.3	–32.8
Recycle compressor	–3.2	–3	–2.6	–3	–3.2	–2.5	–2.8	–3	–2.5	–3.3
Water pumps	–2.9	–3	–3	–3	–3.1	–3.1	–3.2	–3.2	–3.2	–5.7
**Total**	–260	–250.3	–242.7	–271.9	–267.1	–262.8	–290	–286.9	–284.2	–264.5
										
**Power production (MW)**										
Gas turbine 1	183.8	188.9	197.8	232.9	239.6	251.9	261.4	268.7	282.8	0
Gas turbine 2	0	0	0	0	0	0	0	0	0	194.1
Recycle expander	10	5.7	2.5	5.9	3.3	1.5	3.4	1.9	0.9	2.2
LP steam turbine	138.4	143.1	146.1	144.6	145.8	151.1	150	151.9	151.6	143.7
IP steam turbine	67.2	69.7	71.5	70.1	70.9	73.8	72.7	73.8	74.1	69.7
HP steam turbine	50	52.3	52.7	52	54.1	53.4	54.2	55.9	55.5	58.2
**Total**	449.4	459.7	470.6	505.5	513.7	531.7	541.7	552.2	564.9	467.9
										
**Net power** output, MW	**189.4**	**209.4**	**227.9**	**233.6**	**246.6**	**268.9**	**251.7**	**265.3**	**280.7**	**203.4**
										
Equivalent natural gas flow rate (kg/s)	66.7	66.0	65.3	65.1	64.6	63.8	64.4	64.0	63.4	66.2
										
**Methanol production efficiency,** equivalent %	**73.4**	**74.2**	**74.9**	**75.2**	**75.7**	**76.7**	**75.9**	**76.5**	**77.2**	**73.9**
CO_2_ specific emission (gCO_2_/MJmethanol)	0	0	0	0	0	0	0	0	0	14.7

The chemical looping based concept, either through GSDR evaluated in the current study or the internally circulating concept evaluated in a previous study,^44^ proves to provide higher efficiency and more environmentally methanol production. The key uncertainty to further investigate is to prove the ability of the two reactor configurations to operate successfully at pressures relevant to the targeted optimal integration revealed by the study. Furthermore, nitrogen impurities in syngas, resulting from possible gas mixing between the oxidation and reduction stages, should be maintained minimal as it was shown to reduce the overall methanol plant efficiency.^44^

## 4 Conclusion

This study experimentally demonstrates that the novel GSDR process could be optimized for integration into GTL processes to maximize their environmental and efficiency benefits. With GSDR integration to GTL processes, the major greenhouse gases (CO_2_ and CH_4_) are converted to syngas used to produce a variety of downstream products, making a great impact on carbon capture, utilization, and sequestration (CCUS). The three-stage nature of the GSDR cycle makes it perfectly suited for efficient integration with GTL and maximized fuel conversion through recycling the GTL off-gases for reducing the oxygen carrier in GSDR. This study investigated the effect of the CO_2_:CH_4_ ratio, steam addition, and pressure on syngas quality and other GSDR process performances. Process simulations were completed in Aspen to show how the GSDR process could be integrated into the MeOH plant.

The experimental results show that by varying the CO_2_:CH_4_ ratio from 0.25 to 2, syngas with a H_2_/CO molar ratio between 1 and 3 was achieved with up to 90% syngas purity suitable for GTL processes. Although carbon deposition was significant for the CO_2_:CH_4_ ratio less than 2, activity and catalyst stabilities were not negatively affected since the cyclic nature of GSDR ensured that all the produced carbon was gasified/combusted in the oxidation stage on the expenses of reduced CO_2_ capture and utilization efficiency. Substituting part of CO_2_ in the feed by steam has minimized carbon deposition while maintaining the desirable syngas quality (H_2_/CO molar ratio) between 1 and 3 suitable for GTL processes.

A high-pressure operation negatively affected the reforming stage performance, showing a rapid deterioration of CH_4_ and CO_2_ conversion with increased pressure. H_2_ selectivity was also negatively affected driven by the excess unconverted CO_2_ that enhances the RWGS to increase CO selectivity. Interestingly, no carbon deposition has been observed at high pressure. Increased temperature may reduce the negative effect of pressure on the reaction kinetics, but it is unlikely that a performance close to equilibrium will be achieved with this specific oxygen carrier at an economically feasible operating temperature, suggesting the need for research on enhancing the catalytic performance of the oxygen carrier.

Finally, the results suggest that there could be enormous benefits to integrate GSDR into gas-to-liquids processes such as improved process efficiency and reduced GHG emission to enhance commercial deployment. This was demonstrated by the process simulations which have shown that the proposed GSDR process could outperform the conventional ATR process as a syngas source for MeOH synthesis, both on efficiency and CO_2_ emission intensity. However, comprehensive process modeling, techno-economics, and parametric studies are needed to fully map out the potentials of the proposed GSDR–GTL process integration.

## Appendix

Tables A1 and B1 are in the Appendix.

**Table A1:** Summary of Main Streams Conditions, Flow Rate, and Compositions Based on Process Flow Diagram for GSDR-Based Plant

					Mole fractions, %									
Stream No.	Temperature, °C	Pressure, bar	Mole flows, kmol/s	Mass flows, kg/s	CH_4_	CO_2_	H_2_O	CO	H_2_	O_2_	N_2_	AR	Methanol	C_2+_
1	37.0	32.0	4.2	73.5	93.1	1.0	0.0	0.0	0.0	0.0	1.6	0.0	0.0	4.3
2	320.0	12.0	1.9	34.5	0.0	0.0	100.0	0.0	0.0	0.0	0.0	0.0	0.0	0.0
3	15.0	1.0	15.0	434.6	0.0	0.0	0.6	0.0	0.0	20.8	77.6	0.9	0.0	0.0
4	600.4	10.0	10.2	216.5	38.9	14.1	43.7	0.2	0.2	0.0	1.1	0.0	0.0	1.8
6	845.0	10.0	16.7	216.5	6.8	4.6	11.1	23.6	53.2	0.0	0.7	0.0	0.0	0.0
7	702.9	10.0	16.7	216.5	6.8	4.6	11.1	23.6	53.2	0.0	0.7	0.0	0.0	0.0
8	949.8	10.0	5.9	159.9	0.0	34.3	62.4	0.6	0.8	0.0	1.9	0.0	0.0	0.0
9	949.8	10.0	4.1	109.7	0.0	34.3	62.4	0.6	0.8	0.0	1.9	0.0	0.0	0.0
10	949.8	10.0	1.9	50.2	0.0	34.3	62.4	0.6	0.8	0.0	1.9	0.0	0.0	0.0
11	989.7	10.0	11.9	334.4	0.0	0.0	0.8	0.0	0.0	0.0	98.0	1.2	0.0	0.0
12	514.0	1.0	11.9	334.4	0.0	0.0	0.8	0.0	0.0	0.0	98.0	1.2	0.0	0.0
13	102.4	1.0	11.9	334.4	0.0	0.0	0.8	0.0	0.0	0.0	98.0	1.2	0.0	0.0
14	102.4	10.0	16.7	216.5	6.8	4.6	11.1	23.6	53.2	0.0	0.7	0.0	0.0	0.0
15	40.0	10.0	14.9	184.9	7.6	5.2	0.7	26.3	59.5	0.0	0.8	0.0	0.0	0.0
21	246.0	50.0	37.3	627.2	25.5	12.8	0.6	11.4	40.0	0.0	2.6	0.0	7.0	0.0
24	220.0	50.0	34.2	627.2	27.8	14.1	0.6	7.9	34.8	0.0	2.9	0.0	12.1	0.0
30	40.0	11.0	4.0	125.2	0.3	2.9	4.7	0.0	0.0	0.0	0.0	0.0	92.1	0.0
34	24.8	11.0	3.6	59.7	31.5	16.1	0.0	8.8	38.8	0.0	3.2	0.0	1.6	0.0
37	102.4	10.0	1.9	50.2	0.0	34.3	62.4	0.6	0.8	0.0	1.9	0.0	0.0	0.0
48	30.0	152.0	0.7	29.3	0.0	91.2	0.0	1.6	2.0	0.0	5.1	0.0	0.0	0.0

**Table B1:** Summary of Main Streams Conditions, Flow Rate, and Compositions Based on Process Flow Diagram for ATR-Based Plant

					Mole fractions, %									
Stream No.	Temperature, °C	Pressure, bar	Mole flows, kmol/s	Mass flows, kg/s	CH_4_	CO_2_	H_2_O	CO	H_2_	O_2_	N_2_	AR	CH_3_OH	C_2+_
1	37.0	32	4.24	73.54	93.0	1.0	0.0	0.0	0.0	0.0	2.0	0.0	0.0	4.0
2	560.0	27	0.92	16.51	0.0	0.0	100.0	0.0	0.0	0.0	0.0	0.0	0.0	0.0
4	152.0	27	2.70	86.01	0.0	0.0	0.0	0.0	0.0	95.0	5.0	0.0	0.0	0.0
5	225.0	27	7.87	176.06	50.0	1.0	12.0	0.0	0.0	33.0	3.0	0.0	0.0	2.0
6	1059.3	25	13.73	176.06	1.0	3.0	11.0	28.0	56.0	0.0	1.0	0.0	0.0	0.0
10	997.1	25	13.73	176.06	1.0	3.0	11.0	28.0	56.0	0.0	1.0	0.0	0.0	0.0
14	178.1	25	13.73	176.06	1.0	3.0	11.0	28.0	56.0	0.0	1.0	0.0	0.0	0.0
15	40.0	25	12.28	149.95	1.0	3.0	0.0	31.0	62.0	0.0	2.0	0.0	0.0	0.0
16	123.4	52	12.28	149.95	1.0	3.0	0.0	31.0	62.0	0.0	2.0	0.0	0.0	0.0
17	73.8	52	37.84	717.45	10.0	13.0	0.0	25.0	39.0	0.0	13.0	0.0	1.0	0.0
20	204.0	52	37.84	717.45	10.0	13.0	0.0	25.0	39.0	0.0	13.0	0.0	1.0	0.0
21	246.0	50	33.54	717.47	11.0	15.0	0.0	21.0	31.0	0.0	14.0	0.0	8.0	0.0
23	204.0	50	33.54	717.47	11.0	15.0	0.0	21.0	31.0	0.0	14.0	0.0	8.0	0.0
24	220.0	50	30.53	717.41	12.0	16.0	0.0	19.0	24.0	0.0	16.0	0.0	13.0	0.0
30	40.0	3	3.86	124.75	0.0	4.0	1.0	0.0	0.0	0.0	0.0	0.0	94.0	0.0
31	50.0	50	25.55	567.46	14.0	18.0	0.0	21.0	27.0	0.0	18.0	0.0	2.0	0.0
34	41.7	14	1.11	25.19	14.0	19.0	0.0	21.0	26.0	0.0	18.0	0.0	2.0	0.0
35	15.0	1	9.17	264.46	0.0	0.0	0.0	0.0	0.0	21.0	78.0	1.0	0.0	0.0
36	577.0	1	10.03	289.65	0.0	6.0	6.0	0.0	0.0	13.0	74.0	0.0	0.0	0.0
37	178.1	1	10.03	289.65	0.0	6.0	6.0	0.0	0.0	13.0	74.0	0.0	0.0	0.0

## Supporting Information Available

Approach used for process modeling for the autothermal reforming process and methanol plants and process conditions, reactions chemistry, process diagram, and how the proposed GSDR and autothermal reforming process are integrated to methanol production (PDF)
