# Supplementary material for: Carbon Capture Utilization and Storage in Methanol Production Using a Dry Reforming-Based Chemical Looping Technology
Source: Energy Fuels. 2022 Jul 19;36(17):9719–35. doi: 10.1021/acs.energyfuels.2c00620 (PMC9446287; doi:10.1021/acs.energyfuels.2c00620)
Supplement: Supplementary file 1 — ef2c00620_si_001.pdf [file ef2c00620_si_001.pdf]

CCUS in methanol production using a dry reforming based chemical looping technology.

Ambrose UGWU[1*], Mogahid Osman[1], Abdelghafour ZAABOUT[2], Shahriar AMINI[3]**

[1] Dept. of Energy and Process Engineering, Norwegian University of Science and Technology, Norway

[2] Process Technology Department, SINTEF Industry, Norway

[3] Dept. of Mechanical Engineering, The University of Alabama, USA

**Ambrose UGWU, ambrose.ugwu@ntnu.no*

** Shahriar AMINI, samini3@ua.edu*

GSDR-Methanol Process Integration

A process simulation of GSDR integration into the syngas source of a methanol process was completed and compared with autothermal reforming (ATR)to show how the GSDR can be integrated into a MeOH process at different temperature and pressure conditions.

1.1 Gas Switching Dry Reforming (GSDR)

The GSDR process has been described in the main text.

1.2 Autothermal Reforming (ATR) Process

Syngas production using the ATR process described in the DOE report [1] was used as the basis for performance comparison with the GSDR-based process (*Figure S1*). The key difference between the GSDR and ATR systems is that the O_2 required for the syngas production in ATR is supplied by an air separation unit (ASU), while GSDR utilizes an oxygen carrier material. In ATR (as in *Figure S2*), natural gas, oxygen (from the ASU), and steam (extracted from the HRSG) are first mixed and pre-heated before entering the ATR unit. The autothermal reactor is composed of two parts, the burner at the top, and the reforming section at the bottom (which contains a Ni-based catalyst). In the burner, a part of the natural gas feed is combusted with O_2 to supply the energy demand required for the reforming reactions inside the reforming tubes. *Table S1* shows the main reactions that occur inside the ATR combustion and catalytic reforming zone. The operating conditions of ATR were selected based on the DOE report[1]: the steam-to-carbon ratio (H_2O/C) was equal to 0.21, the oxygen-to-carbon ratio (O_2/C) was equal to 0.59, ATR temperature was 1059 °C, and ATR pressure was 25 bar. The ASU was

not modeled in this study, but the electricity consumption was considered to be equal to 0.41 kWh/kg_{O2} similar to the baseline case of the DOE report[1].

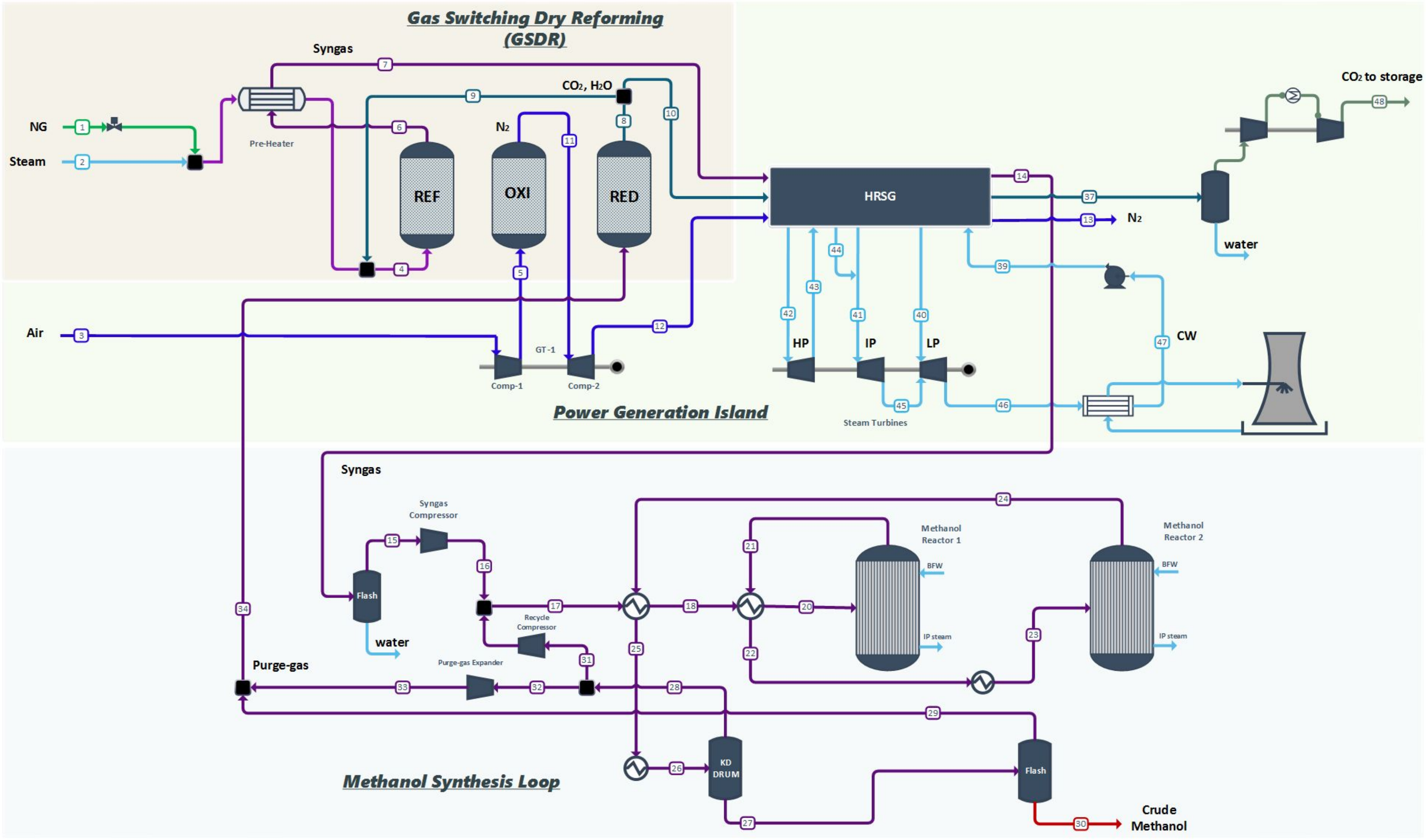

Figure S1. Process flow diagram of GSDR-based methanol production plant

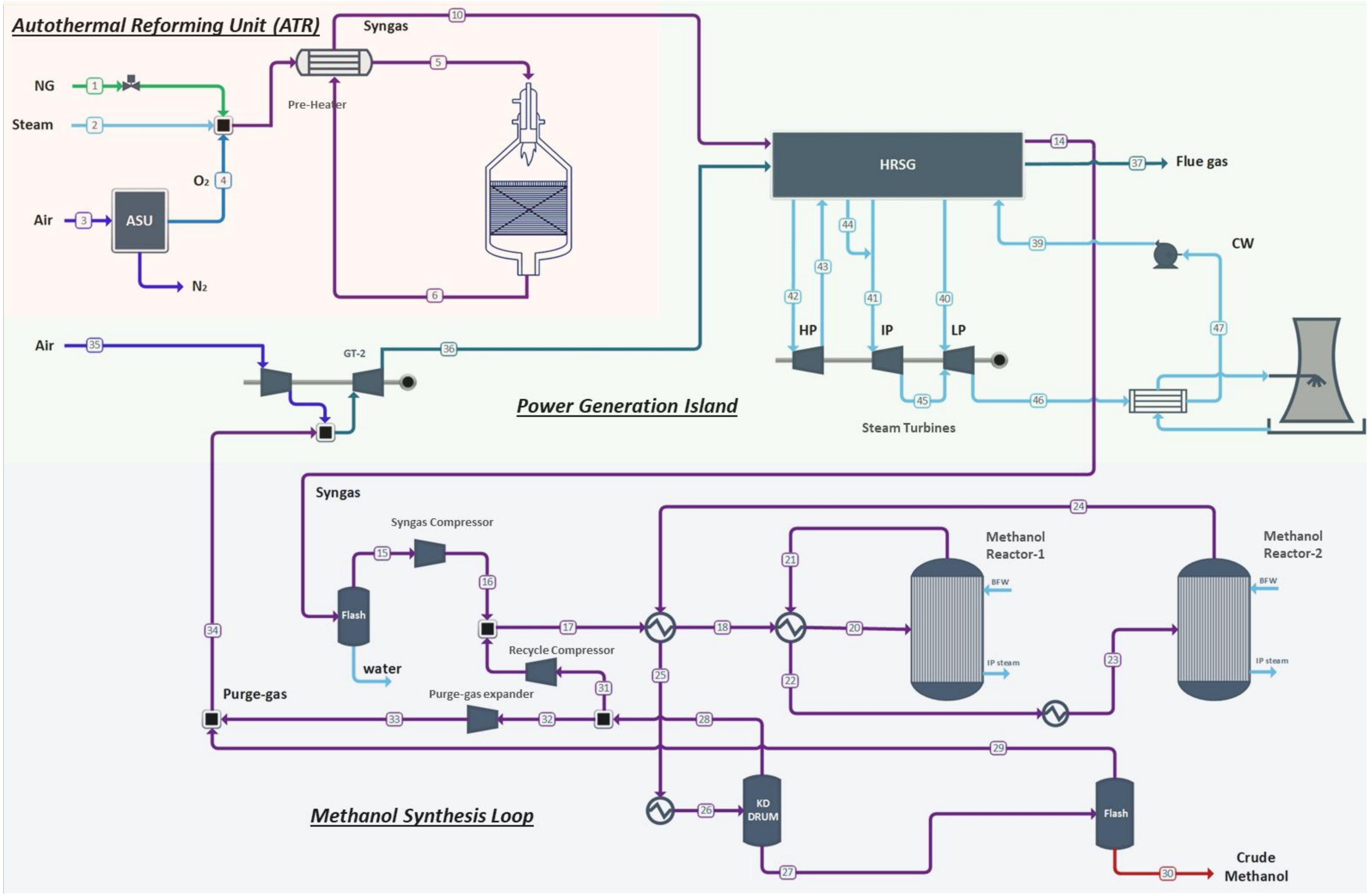

Figure S2. Process flow diagram of ATR-based methanol production plant[2]

Table S1. Summary of the various reactions involved during the ATR process[2].

Reactions	ΔH_{1173K} (kJ/mol)	ΔG_{1173K} (kJ/mol)	Reaction No
Combustion Zone			
$CH_4 + 1.5\ O_2 \leftrightarrow CO + 2H_2O$	-520	-620	(S1)
$CH_4 + O_2 \leftrightarrow CO_2 + 2H_2$	-305	-434	(S2)
$CH_4 + 0.5\ O_2 \leftrightarrow CO + 2H_2$	-23	-254	(S3)
Reformer Zone			
$CH_4 + H_2O \leftrightarrow CO + 3H_2$	226	-70	(S4)
$CO + H_2O \leftrightarrow CO_2 + H_2$	-33	2.6	(S5)

1.3 Methanol Synthesis Loop

The methanol synthesis loop was modelled according to the reference case provided in the DOE report [1]. To achieve high methanol yield, high carbon conversion, and high overall thermal efficiency; the syngas produced through GSDR, and ATR should satisfy the following conditions: 1) The molar ratio H_2/CO should be ~2.0. 2) The stoichiometric number (M) defined as $M = (H_2 – CO_2)/(CO + CO_2)$, should be between 1.7 to 2.0. 3) High CO/CO_2 ratio, as it enhances methanol synthesis reaction rate and boosts the per-pass conversion efficiency as well as decreases the water formation (a typical syngas composition should contain 4 to 8% CO_2 for maximum activity and selectivity). 4) Water content should be minimized because a high steam concentration in the syngas affects the methanol reactor outlet composition through the WGS reaction, and a high amount of water will increase the duty required in the distillation system for the crude methanol purification. 5) The concentration of inert gaseous such as CH_4 and N_2 should be minimized because a high inert gas concentration will lower the partial pressure of the active reactants leading to lower conversion efficiency and hence larger equipment/line size. The operating conditions of GSDR and ATR in this study were adjusted to satisfy all these requirements. The methanol synthesis loop was identical for both plants.

As shown in Figure S1, the methanol synthesis loop consists of various components including two series methanol reactors, heat exchangers, syngas compressor, recycled gas compressor,

flash, and knock-out drum vessel. The methanol reactors are catalytic packed-beds with a copper-based catalyst. *Table S2* shows the reactions that take place during the syngas conversion to methanol.

As it can be seen from *Table S2*, reactions (S6 and (S7 are exothermic and involve a decrease in the number of moles, which means that high pressure and low-temperature operation is essential to shift the equilibrium towards methanol production. Therefore, the methanol reactors are operated in a temperature range of 220 – 250 °C and a pressure of 50 bar. To achieve near isothermal operation and hence maximize the per-pass conversion to methanol, the reactors are continually cooled down using

Table S2. Summary of the reactions involved during methanol synthesis.

**Reactions**	**$\Delta H_{298K, 50bar}$ (kJ/mol)**	**Reaction No**
$CO + 2\,H_2 \leftrightarrow CH_3OH$	-91	(S6)
$CO_2 + 3\,H_2 \leftrightarrow CH_3OH + H_2O$	-41	(S7)
$CO_2 + H_2 \leftrightarrow CO + H_2O$	-50	(S8)

a boiling water stream flowing through the shell side of the reactors. The produced steam at intermediate pressure is sent to the HRSG for superheating and integration with the power cycle.

Given the thermodynamic equilibrium limitation, the conversion per-pass is expected to be low (typically around 25%), therefore, a large recycle of unconverted gas is sent back to the methanol reactors after re-compression and heating. The continual recycling of the syngas results in a build-up of the inert gases (e.g., N_2, and CH_4) in the system (as they do not involve in the methanol synthesis reactions), therefore, around 4% of the recycled gas is purged out of the loop and burnt as fuel for power generation in a combined cycle. The rich methanol stream (stream 27) is cooled down, and the liquid is separated in the last flash vessel, to produce the crude methanol. The crude methanol is then sent to distillation columns for further purifications to 99.9% (the final purification step was not considered in the current study).

1.4 Power Generation Island and HRSG

Power generation in the ATR and GSDR-based methanol plants was achieved using a Brayton-Rankine combined cycle. The generated power aimed to make the plant self-sufficient for energy supply as well as to export extra power to the local electricity grid. The GSDR-based plant uses a gas turbine that utilizes the pressurized exhaust gas from the OXI stage, while the ATR-based plant uses a gas turbine based on purge gas combustion. The Heat Recovery Steam Generator (HRSG) utilizes the heat of different streams and units within the process for steam production including syngas produced from GSDR and ATR, exhaust gas from gas turbine 1, exhaust gas from gas turbine 2, and the methanol synthesis reactors. The generated steam is expanded in a triple pressure steam cycle, and part of the steam is used for the reforming reactions in GSDR and ATR (*Table S1*) shows the design specification of the steam turbines. The HRSG was modelled using a series of heat exchangers consisting of economizers, evaporators, and super-heaters, which were designed to extract the maximum recoverable heat from the process.

References

1. Spallina, V., et al., *Techno-economic assessment of an integrated high pressure chemical-looping process with packed-bed reactors in large scale hydrogen and methanol production.* International Journal of Greenhouse Gas Control, 2019. **88**: p. 71-84.
2. Osman, M., et al., *Pressurized chemical looping methane reforming to syngas for efficient methanol production: Experimental and process simulation study.* Advances in Applied Energy, 2021. **4**: p. 100069.
